# Potassium-driven coordination of carbon and nitrogen metabolism to enhance cotton fiber development under limited drip irrigation conditions

**DOI:** 10.3389/fpls.2026.1946477

**Published:** 2026-09-25

**Authors:** Xiaojuan Shi, Liwen Tian, Nannan Li, Feng Shi, Yu Tian, Hongxia Zhang, Houxiu Zhao, Jun Wang, Xianzhe Hao, Honghai Luo

**Affiliations:** 1 College of Agriculture, Shihezi University, Shihezi, Xinjiang, China; 2 Cotton Research Institute of Xinjiang Uygur Autonomous Region Academy of Agricultural Sciences/National Cotton Engineering Technology Research Centre, Urumqi, Xinjiang, China; 3 Xinjiang Academy of Agricultural and Reclamation Science, Shihezi, Xinjiang, China; 4 Key Laboratory of Northwest Oasis Water−Saving Agriculture, Ministry of Agriculture and Rural Affairs, Shihezi, Xinjiang, China

**Keywords:** carbon and nitrogen metabolism, cellulose accumulation, cotton fiber synthesis, limited drip irrigation, potassium

## Abstract

**Introduction:**

Cotton is the world’s dominant natural fiber crop, and its production in arid oasis systems is widely constrained by drought. Potassium supplementation can alleviate water-deficit damage in cotton. However, the core mechanism linking potassium signaling to fiber carbon–nitrogen coordination and cellulose biosynthesis remains uncharacterized fiber.

**Methods:**

Field experiments were conducted from 2021 to 2023 under two distinct drip irrigation regimes-conventional (500 mm, I_500_) and limited (425 mm, I_425_)—and three different potassium fertilization levels (low-potassium K_1_: 48 kg ha^-1^; medium-potassium K_2_: 144 kg ha^-1^; and high-potassium K_3_: 240 kg ha^-1^). Key enzyme activities, metabolite contents, cellulose accumulation levels, and cotton fiber carbon and nitrogen metabolism yields were measured.

**Results:**

Under limited drip irrigation conditions, the activities of sucrose synthase (SS), sucrose phosphate synthase (SPS), and β-1,3-glucanase increased by 11.1–21.2%, 9.9–21.4%, and 5.8–27.2%, respectively, in the medium-potassium treatment (I_425_K_2_). These changes promoted the accumulation of sucrose and β-1,3-glucan during the critical period of fiber development and provided sufficient substrates for cellulose synthesis. Concurrently, the activities of nitrate reductase (NR) and glutamine synthetase (GS) increased by 9.7–33.2% and 7.3–30.1%, respectively, increasing the free amino acid and soluble protein contents and optimizing nitrogen metabolism. This treatment significantly reduced the fiber sucrose-to-soluble-protein (S/P) ratio during the secondary wall thickening period (40–50 days), indicating a more coordinated allocation between carbon substrates and nitrogenous metabolites in developing fibers. The cellulose content increased by 22.7% compared with that in the I_500_K_1_ treatment, and the fiber biomass and lint percentage significantly increased. Ultimately, the I_425_K_2_ treatment achieved the highest three-year mean lint yield, representing a 26.0% increase over the lowest-yielding I_500_K_1_ treatment, and simultaneously improved irrigation water use efficiency (IWUE) by 39.6% relative to I_500_K_1_. The I_425_K_3_ treatment ranked second in lint yield. Multivariate correlation, Mantel test and principal component analyses further showed that coordinated carbon/nitrogen enzyme activity, a reduced S/P ratio, and enhanced cellulose accumulation were associated with elevated lint yield and IWUE.

**Discussion:**

We conclude that moderate potassium fertilization under regulated limited drip irrigation synergistically activates core carbon- and nitrogen-metabolic enzymes, optimizes the balance between fiber sucrose supply and soluble protein availability, and accelerates cellulose synthesis to simultaneously increase fiber biomass and lint output. This water-potassium co-regulation framework delivers a field-deployable, water-saving high-yield agronomic strategy tailored to arid cotton-growing regions, and establishes a mechanistic foundation for optimizing source-sink carbon-nitrogen partitioning under chronic water limitation.

## Highlights

- Moderate potassium supply synchronously upregulates fibre carbon and nitrogen rate-limiting enzymes under deficit drip irrigation, balancing fibre C/N homeostasis during secondary wall thickening.
- Coordinated carbon–nitrogen metabolism elevates cellulose precursor pools and boosts cellulose deposition by 26.3%, accelerating cotton fibre biomass accumulation.
- Limited drip irrigation with medium potassium improves lint yield and water use efficiency in arid regions.

## 1 Introduction

As the most important natural fiber crop worldwide, cotton is directly linked to the economic benefits and sustainable development of the textile industry (Wang et al., 2022). The primary cotton-producing regions are predominantly located in arid and semiarid areas, making cotton production highly susceptible to drought stress. Under the backdrop of climate change, drought has become a critical environmental factor limiting cotton yield improvement and high-quality fiber formation (Wei et al., 2018; Liu et al., 2024). Drought stress impedes cotton growth, disrupts its physiological metabolism, and ultimately significantly reduces its yield and quality. Therefore, identifying effective strategies to alleviate drought damage to cotton while promoting efficient use of limited water is essential. Such strategies are central to ensuring sustainable cotton production in arid and semiarid regions.

Sucrose metabolism plays a central role during fiber development, ultimately leading to reduced fiber biomass and cellulose contents (Hu et al., 2017). Drought stress inhibits the activity of key enzymes such as sucrose phosphate synthase (SPS) and sucrose synthase (SS) (Chantal et al., 2007), thereby impeding sucrose breakdown and resulting in an insufficient supply of uridine diphosphate-glucose (UDPG), which is a precursor for cellulose synthesis (Alsherif et al., 2022; Hu et al., 2022). This process disrupts the dynamic balance between β-1,3-glucanase-mediated callose degradation and cellulose deposition (Tucker et al., 2001; L. Liu et al., 2023). Drought stress typically restricts cotton growth and fiber development by suppressing photosynthesis, carbon and nitrogen metabolism, and assimilate partitioning (Sakoda, 2021). However, not all water deficits inevitably reduce fiber yield. Moderately limited water deficits may induce physiological compensation effects. Therefore, exploring the optimization of cultivation management practices (e.g., precise nutrient regulation) to stimulate compensation effects under limited water deficit conditions, thereby stabilizing or even increasing yield, is a core strategy for achieving the synergistic goals of water conservation and yield improvement in cotton production.

Potassium (K) nutrition regulation has been proven to be an effective strategy for alleviating drought stress and optimizing carbon–nitrogen metabolism to increase cotton yield and fiber quality (Zahoor et al., 2017a). Potassium (K^+^), the dominant cation in plant tissues, serves as an essential activator for more than 60 enzymes. Potassium significantly enhances the stomatal regulation capacity, osmotic protection, and antioxidant defense system of cotton, thereby improving overall drought resistance (Kumar et al., 2020; Johnson et al., 2022). Additionally, potassium directly promotes the activity of key enzymes involved in sucrose metabolism, such as SPS and SS (Ju et al., 2023), driving the efficient decomposition of sucrose into UDPG and guiding its preferential utilization for cellulose synthesis rather than for callose accumulation (Tucker et al., 2001). Moreover, potassium helps maintain the coordination between carbon and nitrogen metabolism, effectively reducing the elevated sucrose-to-protein ratio caused by stress (Hu et al., 2017) and thereby mitigating the adverse effects of drought on fiber development. However, the specific physiological mechanisms by which potassium regulates the aforementioned processes (particularly the activity of key enzymes involved in sucrose metabolism and cellulose synthesis) to synergistically influence cotton fiber yield under limited drip irrigation conditions (i.e., limited water deficit) require further investigation.

In 2023, the total global cotton production reached 2470.0×10^4^ tons, and China’s total cotton production accounted for 22.7% (561.80×10^4^ tons) of the global total cotton production in the same year (USDA, 2023). China is a major producer and consumer of cotton worldwide. With the advantages of integrated drip irrigation technology and light resources (Lin et al., 2024), the Xinjiang cotton region has become a major cotton production base in China. Owing to the typical continental arid climate and oasis irrigation agriculture characteristics of China, water resources must be efficiently utilized to ensure the sustainable development of the cotton industry. Drip irrigation technology provides a foundation for implementing precise integrated water and fertilizer management, such as by integrating limited drip irrigation with K fertilizer management practices. Our research group revealed that in the early stage, increasing the K fertilizer application rate appropriately in arid or semiarid areas with a limited water supply promotes the development of fiber and cottonseed, resulting in a simultaneous improvement in cotton yield and fiber quality. Our research group revealed that in the early stage, increasing the K fertilizer application rate appropriately in arid or semiarid areas with a limited water supply promotes the development of fiber and cottonseed, resulting in a simultaneous improvement in cotton yield and fiber quality. Nevertheless, most existing water–potassium interaction studies on cotton have been conducted under pot conditions or within a single growing season, which cannot eliminate interannual climatic fluctuations (e.g., spring chilling and late-season rainfall) and may overestimate or underestimate the stability of fertigation effects. A multi-year fixed-location field trial is therefore required to generate repeatable physiological and agronomic evidence for arid cotton production. On the basis of these findings, we propose a three-tier scientific hypothesis. Under limited drip irrigation, appropriate potassium management will (1) synergistically activate key enzymes of sucrose and nitrogen metabolism (SS, SPS, β-1,3-glucanase, NR and GS); (2) optimize the fiber sucrose-to-soluble-protein (S/P) ratio and thereby support the carbon precursor supply required for cellulose polymerization; and (3) accelerate fiber development and thereby improve cotton lint yield and water productivity.

Therefore, in this study, the physiological mechanisms through which the UDPG supply and cellulose synthesis affect cotton fiber development are explored to determine the effects of limited drip irrigation and potassium coregulation on the balance between fiber carbon and nitrogen metabolism and the efficiency of cellulose synthesis. The key objectives of this research are to (1) explore the physiological mechanisms by which potassium regulates carbon and nitrogen metabolism under limited drip irrigation conditions, thereby affecting the UDPG supply and cellulose synthesis, and (2) elucidate the correlation between the coordination of fiber carbon and nitrogen metabolism (indexed by the sucrose-to-soluble-protein ratio) and fiber yield formation as water and potassium interact and provide a theoretical basis for achieving high cotton yield and quality in arid or semiarid areas.

## 2 Materials and methods

### 2.1 Experimental site and cultivars

The field experiment was conducted from 2021 to 2023 at the Crop Efficient Water Use Observation Station at 45°38’N, 86°09’E, Xinjiang Academy of Agricultural Reclamation Sciences. The tested cotton variety (*Gossypium hirsutum* L.) was Xinshi K21, whose growth period was 121 days; the cotton was obtained from the Shihezi Agricultural Science Research Institute. The meteorological data for the cotton growing season included the daily average temperature, maximum temperature, minimum temperature, and total precipitation (**Figure 1**). The basic physical and chemical properties of the 0–20-cm soil layer were as follows: organic matter, 15.00 g kg^-1^; total nitrogen, 1.05 g kg^-1^; alkali-hydrolyzable nitrogen, 42.20 mg kg^-1^; available phosphorus, 19.81 mg kg^-1^; and available potassium, 274.28 mg kg^-1^.

**Figure 1:**
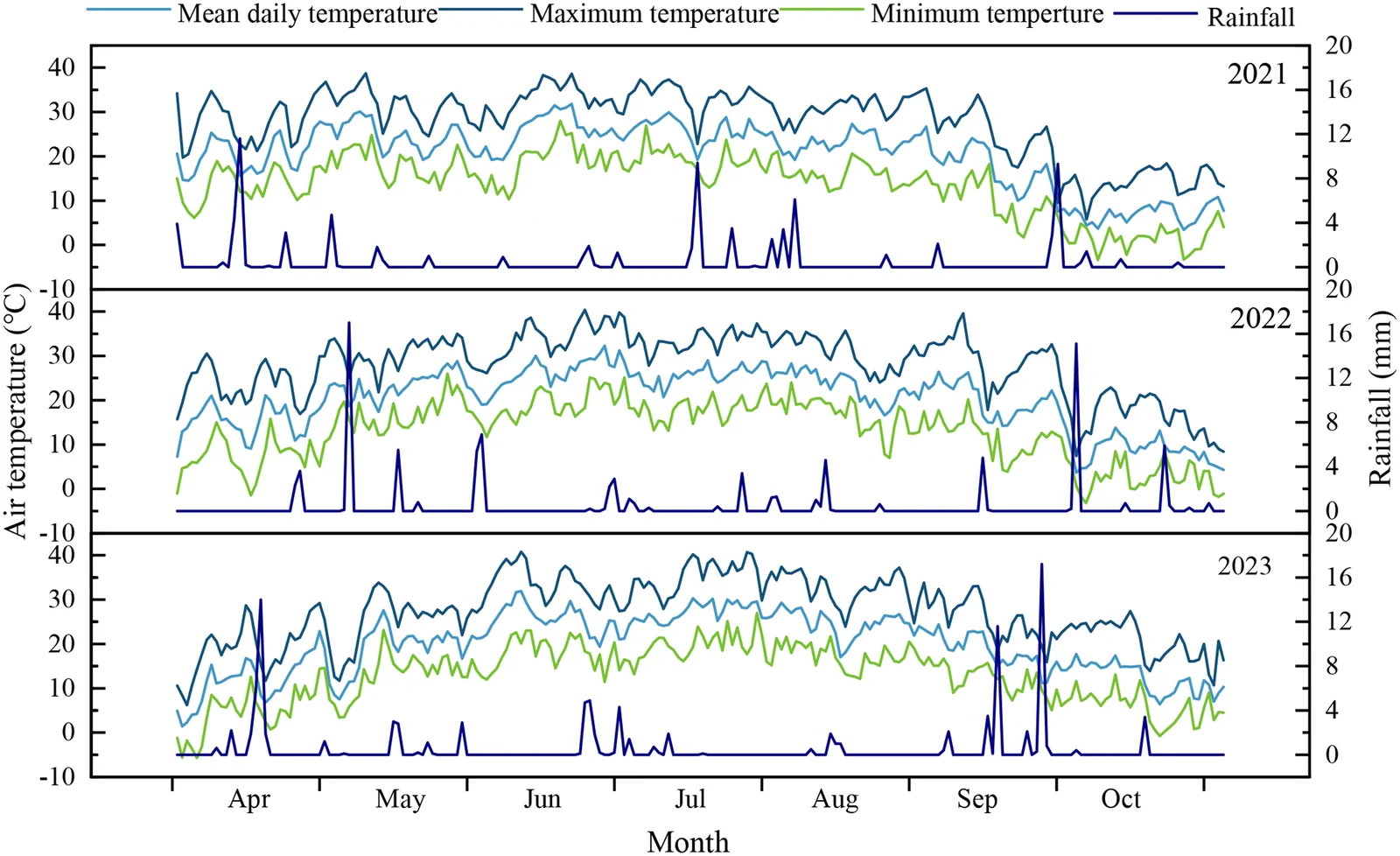
Meteorological data during the cotton growing season.

### 2.2 Experimental design and field management

For the experiment, we adopted a two-factor split zone experimental design. The main area was for drip irrigation, and the subarea was for potassium application. Two types of drip irrigation rates were used: conventional (500 mm, I_500_) and limited (425 mm, I_425_). Limited drip irrigation reduced the drip irrigation rate by 38.0% and 15.0% during the bud and opening stages, respectively, under mild water stress conditions, whereas the same drip irrigation rate was maintained for I_500_ and I_425_ during the flowering and boll stages (Niu et al., 2016). The three potassium application levels were low potassium (K_1_, 48 kg ha^-1^), medium potassium (K_2_, 144 kg ha^-1^), and high potassium (K_3_, 240 kg ha^-1^), as reported by Tang et al (Tang et al., 2022). The application ratios of potassium fertilizer (K_2_O) during the cotton growth period were as follows: bud:flowering:boll opening = 15.0%:79.0%:6.0%. The size of each plot was 68.4 m^2^ (10 m×6.84 m), and all the treatments were replicated three times, resulting in a total of 18 experimental units. The irrigation and fertilization plans are shown in **Table 1**.

**Table 1:** Irrigation (mm) and fertilization strategies (kg ha^-1^).

Treatment		Squaring	Flowering	Boll	Total
Irrigation pattern	Conventional drip irrigation (I_500_, mm)	173.0	270.0	57.0	500
	Limited drip irrigation (I_425_, mm)	106.5	270.0	48.5	425
Potassium	K_1_ (K_2_O, kg ha^-1^)	11.5	29.3	7.2	48
	K_2_ (K_2_O, kg ha^-1^)	34.6	87.8	21.6	144
	K_3_ (K_2_O, kg ha^-1^)	57.6	146.4	36.0	240
Nitrogen	N (kg ha^-1^)	50.4	172.8	16.8	240
Phosphate	P_2_O_5_ (kg ha^-1^)	23.4	97.5	9.1	130

The sources of N, P_2_O_5_ and K_2_O were urea (46.0% N), ammonium dihydrogen phosphate (12.0% N, 61.0% P_2_O_5_), and potassium sulphate (50.0% K_2_O). The application rates of N and P_2_O_5_ were 240 kg ha^-1^ and 130 kg ha^-1^, respectively (Tian et al., 2023), and all fertilizers were applied via topdressing with water droplets. We followed locally recommended cotton production practices for all other field management practices. In 2021, the crop was sown on April 24 and harvested on September 30. The 2022 growing season ran from April 24 to October 16, while in 2023, sowing occurred on May 2, and harvesting occurred on October 9. The planting method consisted of a wide narrow row, with one membrane, three tubes, and six rows (**Figure 2**). The average planting density from 2021 to 2023 was 258244 plants per hectare.

**Figure 2:**
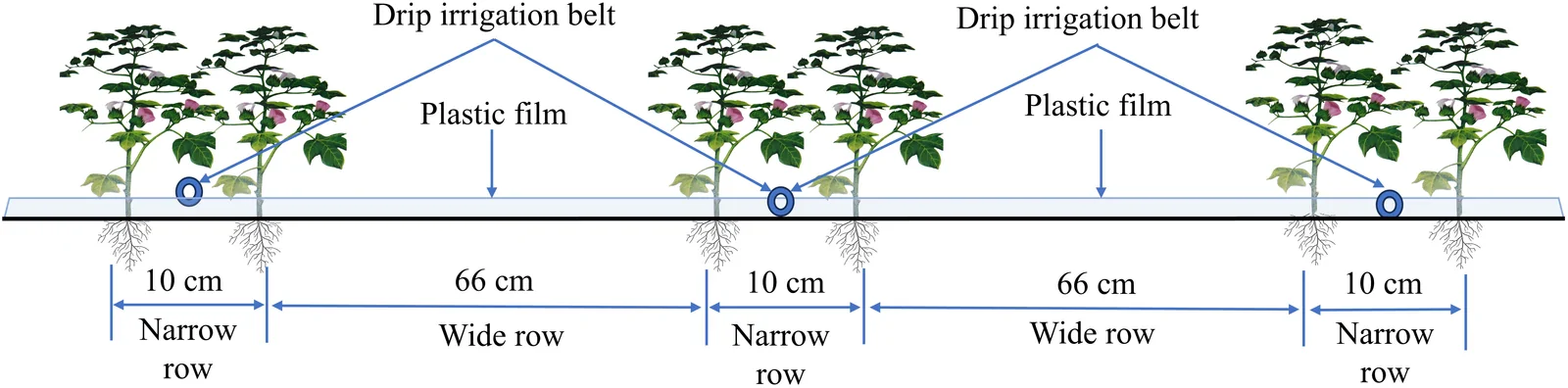
Schematic diagram of the planting pattern (2021–2023).

### 2.3 Measurement items and methods

Sampling protocol: At the first flowering day, white flowers at the first fruiting node of designated branches were tagged. For all biochemical and molecular analyses (enzyme activities, metabolite contents, cellulose content), bolls were sampled at 20, 30, 40, and 50 days post-anthesis (DPA). Specifically, bolls from the first fruiting node of the 3rd–4th (lower canopy) and 6th–7th (upper canopy) fruiting branches were collected. At each sampling time point, six uniformly developed bolls per plot were harvested between 9:00 and 10:00 a.m. The fibers were carefully separated from the seeds, immediately frozen in liquid nitrogen, and stored at −80 °C. For each plot, fibers from the six bolls were pooled into one biological replicate, yielding three biological replicates per treatment (one per replication block). For fiber biomass determination (Section 2.3.3), bolls were sampled every 10 days from 10 to 50 DPA using the same branch positions and pooling strategy.

#### 2.3.1 Evaluation of carbon and nitrogen metabolites in cotton fiber and β-1,3-glucan content in cotton fibers

The sucrose content was determined using the anthrone–sulfuric acid method (Zhang et al., 2020). Dried fibers (0.5 g) were extracted twice with 10 mL of 80% ethanol at 80 °C for 30 min, which was followed by centrifugation at 8000 g for 15 min. The combined supernatants were evaporated at 50 °C and diluted to 50 mL with distilled water. Afterwards, 1.0 mL of extract was reacted with 2.0 mL of 0.2% anthrone in concentrated sulfuric acid in a boiling water bath for 7 min. After cooling, the absorbance was measured at 620 nm. The sucrose content was calculated using a sucrose standard curve and expressed as mg·g^-1^ DW.

The β-1,3-glucan content was assayed according to the methods of Shu et al. (2009) with modifications. Dried fibers (0.15 g) were extracted twice with 5 mL of 50 mM citrate–phosphate buffer (pH 6.5) in a boiling water bath for 15 min. After centrifugation (8000 g, 10 min), the supernatants were pooled until a volume of 20 mL was reached. The extract (100 μL) was incubated with lichenase (EC 3.2.1.73) at 40 °C for 60 min, followed by β-glucosidase (EC 3.2.1.21) at 40 °C for 30 min. Glucose release was determined with GOPOD reagent at 510 nm. The β-1,3-glucan content was calculated as (total glucose – free glucose) × 0.9 and expressed as mg·g^-1^ DW.

The soluble protein content was measured using the BCA method (Liu et al., 2023). Fresh fibers (0.1 g) were homogenized in 1 mL of precooled PBS (50 mM, pH 7.0) and centrifuged at 12000 g at 4 °C for 10 min. The supernatant (5 μL) was mixed with 200 μL of BCA working solution and incubated at 60 °C for 30 min. The absorbance was measured at 562 nm. The protein content was determined using a BSA standard curve and expressed as mg·g^-1^ FW.

The fiber sucrose-to-soluble-protein ratio (S/P ratio) was calculated by dividing the sucrose activity (expressed on a dry-weight basis, mg g^-1^ DW) by the soluble protein content (expressed on a fresh-weight basis, mg g^-1^ FW). Because the numerator and denominator were quantified on different weight bases and represent only a specific carbon-containing metabolite and a nitrogen-containing metabolite, respectively, this ratio is not a true elemental carbon-to-nitrogen ratio and is hereafter referred to as the S/P ratio throughout the manuscript.

The free amino acid content was determined using an ELISA-based assay (Liu et al., 2023). Fresh fibers (0.2 g) were extracted with 4 mL ultrapure water at 80 °C for 30 min, followed by centrifugation (12000 × g, 15 min). The supernatant was collected and diluted to 15 mL. Afterwards, 10 μL of diluted extract was mixed with 40 μL of sample diluent and 50 μL of assay reagent on an ELISA plate. After incubation at 37 °C for 60 min and washing, color developers A and B were added. The reaction was stopped after 15 min, and the absorbance was read at 450 nm. The free amino acid content was calculated using an L-glutamic acid standard curve and expressed as mg·g^-1^ FW.

#### 2.3.2 Extraction and determination of key enzyme activity and cellulose content in cotton fibers

The sampling method for enzyme activity analysis was the same as that described in Section 2.3.1. For cellulose determination, representative cotton bolls were collected at 20, 30, 40, and 50 days of age and at harvest. Bolls from the first fruit node of the 3rd to 4th and the 7th to 8th fruiting branches were selected. The fibers were separated, immediately frozen in liquid nitrogen, and stored at -80 °C for subsequent analysis.

The activities of sucrose synthase (SS, U·g^-1^ FW) and sucrose phosphate synthase (SPS, U·g^-1^ FW) were determined. The extraction and activity assays of SS in the synthetic direction and SPS were performed following the methods of Zhao et al. (2020) after optimization. Cotton fiber samples (0.5 g) stored at -80 °C were ground thoroughly in 5 mL of precooled extraction buffer in an ice bath. The extraction buffer consisted of 50 mM HEPES–NaOH (pH 7.5), 10 mM MgCl_2_, 1 mM EDTA, 10 mM DTT, 10 mM ascorbic acid, and 2% (w/v) insoluble polyvinylpolypyrrolidone (PVPP). The homogenate was subsequently centrifuged at 12,000 × g for 20 minutes at 4 °C. The resulting supernatant, designated the crude enzyme extract, was used for assaying the activity of SS and SPS. The soluble protein content in the supernatant was determined by the Bradford method.

For the SPS activity assay, the reaction mixture (total volume of 200 µL) contained 50 mM HEPES–NaOH (pH 7.5), 15 mM MgCl_2_, 10 mM fructose-6-phosphate, 30 mM glucose-6-phosphate, 15 mM UDPG, and 100 µL of enzyme extract. The mixture was incubated in a water bath at 30 °C for 30 minutes, and the reaction was immediately terminated by placing the tubes in a boiling water bath for 3 minutes. After cooling, 200 µL of 40% (w/v) KOH solution was added, and the tubes were heated in a boiling water bath for 10 minutes to destroy the unreacted fructose and fructose-6-phosphate. Then, after cooling, 3 mL of 0.15% (w/v) resorcinol (dissolved in 95% ethanol) and 9 mL of 30% HCl were added. The mixture was vortexed and incubated in a water bath at 80 °C for 10 minutes for color development. After cooling, the absorbance was measured at 480 nm. A standard curve was prepared using different concentrations of sucrose standards subjected to the same treatment.

For the SS activity assay (synthesis direction), the reaction mixture (total volume of 200 µL) contained 50 mM HEPES–NaOH (pH 7.5), 15 mM MgCl_2_, 25 mM fructose, 15 mM UDPG, and 100 µL of enzyme extract. The incubation and termination steps were the same as those for the SPS assay, but the KOH boiling step was omitted. Because the substrate was fructose, the produced sucrose had to be preserved. The resorcinol–HCl reagent was added directly for color development, and the absorbance was measured at 480 nm.

The activity of β-1,3-glucanase (EC 3.2.1.39) was assayed using the 3,5-dinitrosalicylic acid (DNS) colorimetric method described by Hu et al. (2017), with laminarin serving as the specific substrate. Frozen fiber samples (0.3 g) were ground in an ice bath with 3 mL of 50 mM acetate buffer (pH 5.0) containing 1 mM EDTA and 5 mM β-mercaptoethanol. The homogenate was centrifuged at 10,000 × g for 20 minutes at 4 °C, and the resulting supernatant was used for the enzyme activity assay. The enzymatic reaction system was prepared as follows. First, 250 µL of appropriately diluted enzyme extract was mixed with 250 µL of 0.5% (w/v) laminarin (dissolved in 50 mM acetate buffer, pH 5.0). The mixture was incubated for precisely 60 minutes in a water bath at 37 °C. The reaction was terminated immediately by the addition of 1.5 mL of DNS reagent, followed by heating in a boiling water bath for 5 minutes for color development. After cooling under running water, the mixture was diluted to a final volume of 10 mL with distilled water, and the absorbance was measured at 540 nm. A control tube was prepared by inactivating the enzyme with DNS before the enzyme extract was added. Then, the same procedure was performed. A standard curve was generated using glucose as a standard. One unit (U) of enzyme activity was defined as the amount of enzyme that catalyzed the production of 1 µmol of glucose, reducing the equivalent per minute under the assay conditions. The results were expressed as U·g^-1^ FW.

Nitrate reductase (NR; EC 1.7.1.1) activity was determined using an *in vitro* assay following the methods of Zahoor et al. (2017a) after performing minor modifications. As NR is a highly unstable substrate-inducible enzyme, all procedures were performed rapidly at 4 °C, and fresh samples or samples stored in liquid nitrogen were used. Frozen fiber samples (0.5 g) were ground with 5 mL of precooled extraction buffer (100 mM potassium phosphate buffer, pH 7.5, 1 mM EDTA, 10 mM cysteine, 10 µM FAD, and 1% (w/v) casein). The homogenate was centrifuged at 10,000 × g for 15 minutes at 4 °C, and the resulting supernatant was used as the crude enzyme extract. The reaction mixture for the enzyme activity assay (total volume of 1.0 mL) consisted of 0.4 mL of 100 mM potassium phosphate buffer (pH 7.5), 0.1 mL of 0.1 M KNO_3_, 0.1 mL of 2 mM NADH (freshly prepared), and 0.4 mL of enzyme extract. The reaction was conducted in a water bath at 25 °C for exactly 30 minutes. Then, the reaction was terminated immediately by adding 1 mL of 1% (w/v) sulfanilamide (dissolved in 3 M HCl). Afterwards, 1 mL of 0.02% (w/v) N-1-naphthylethylenediamine dihydrochloride was added for color development. After standing at room temperature for 15 minutes, the mixture was centrifuged at 4,000 × g for 15 minutes at 4 °C to remove turbidity. The absorbance of the supernatant was measured at 540 nm. A control tube was prepared by adding the sulfanilamide terminator before the enzyme extract, and the same procedure was performed again. A standard curve was generated using sodium nitrite as a standard. NR activity was expressed either in the form of micrograms of nitrite produced per gram of fresh weight per hour (µg NO_2_^-^·g^-1^ FW·h^-1^) or on a protein basis.

Glutamine synthetase (GS; EC 6.3.1.2) activity was assayed using a modified method based on the γ-glutamyltransferase reaction (Zahoor et al., 2017a). Frozen fiber samples (0.5 g) were ground with 4 mL of precooled extraction buffer (50 mM imidazole–HCl buffer, pH 7.2, 10 mM MgSO_4_, 1 mM EDTA, 5 mM DTT, and 1% (w/v) PVPP). The homogenate was centrifuged at 12,000 × g for 25 minutes at 4 °C, and the supernatant was used for the assay. The reaction mixture (total volume of 1.6 mL) consisted of 0.5 mL of 250 mM imidazole–HCl buffer (pH 7.2), 0.2 mL of 300 mM glutamine, 0.2 mL of 300 mM Na_2_HA_S_O_4_, 0.2 mL of 60 mM MnCl_2_, 0.2 mL of 40 mM ADP (pH 7.0), 0.2 mL of 2 M hydroxylamine (freshly prepared and neutralized to pH 7.0 with NaOH), and 0.1 mL of enzyme extract. After the reaction was started, the mixture was incubated in a water bath at 37 °C for 30 minutes. The reaction was terminated, and the color was developed by adding 1.0 mL of stop solution and color development solution (0.37 M FeCl_3_, 0.67 M HCl, and 0.2 M trichloroacetic acid). The reaction mixture was centrifuged at 3,000 × g for 10 minutes at 4 °C to remove the precipitate, and the absorbance of the supernatant was measured at 540 nm. A control tube was prepared using boiled inactivated enzyme extract. A standard curve was generated using L-glutamic acid γ-monohydroxamate as a standard. One unit (U) of GS activity was defined as the amount of enzyme catalyzing the formation of 1 µmol of γ-glutamyl monohydroxamate per minute under the assay conditions. The results were expressed as U·g^-1^ FW protein.

The cellulose content in the cotton fibers was determined using a modified version of the method of Hu et al. (2022), in which hemicellulose and lignin were effectively removed, thereby improving the accuracy. Fiber samples were first dried to a constant weight at 80 °C and then ground. An accurately weighed 50 mg dry sample was placed into a screw-capped tube, and 5 mL of acetic–nitric acid reagent (nitric acid:glacial acetic acid:distilled water = 1:8:1, v/v/v) was added. The tube was capped tightly and heated in a boiling water bath for 30 minutes with intermittent shaking. This step removed lignin, hemicellulose, and starch. After the reaction, the tube was cooled and centrifuged at 3,000 × g for 10 minutes, and then the supernatant was discarded. The precipitate was washed three times with distilled water; after each wash, centrifugation was performed and supernatant was discarded. Finally, 5 mL of 67% (v/v) sulfuric acid (prepared by mixing concentrated sulfuric acid with water proportionally, with caution) was added to the precipitate, and the mixture was allowed to stand at room temperature for 1 hour with intermittent shaking to completely dissolve the cellulose. The dissolved solution was appropriately diluted. An aliquot of 1.0 mL of the diluted solution was mixed with 5.0 mL of freshly prepared 0.2% anthrone reagent (dissolved in concentrated sulfuric acid) and heated in a boiling water bath for exactly 10 minutes. After cooling, the absorbance was measured at 620 nm. A standard curve was generated using cellulose standards (Sigma–Aldrich) subjected to the same treatment. The cellulose content was expressed as a percentage of the dry weight (% DW) of the sample.

#### 2.3.3 Single boll fiber biomass

When the lower part (3–4 branches) and upper part (6–7 branches) of the cotton plant bloomed at the first fruiting node, the white flowers that bloomed on the day of labelling were marked, and the bell period was initiated. Starting from a boll age of 10 days (the 10th day of lower boll age corresponded to day 0 of the upper cotton bolls), cotton boll samples were collected every 10 days until 50 days of age. From 9:00 to 10:00 in the morning, 6 cotton bolls whose growth and development were consistent were collected and transferred to the laboratory. The fibers of the cotton bolls were separated and air dried to a constant weight, and data were recorded.

The process of fiber biomass accumulation was described using a logistic equation (Yang et al., 2011):


$$\text{y}=\frac{\text{K}}{1+{\text{ae}}^{\text{bt}}}$$

In the formula, y is the fiber biomass corresponding to the boll age (g boll^-1^); K is the maximum fiber biomass (g boll^-1^); t is the age of the boll (d); and a and b are constants.

The starting time (*t_1_*, d) and ending time (*t_2_*, d) of the rapid period of fiber biomass accumulation were determined as follows:


$${\text{t}}_{1}=\;\frac{1}{b}\ln(\frac{2+\sqrt{3}}{a}),t_{2}=\frac{1}{b}\ln(\frac{2-\sqrt{3}}{a})$$

The rapid accumulation period of the fiber biomass T (d) started at *t_1_* and ended at *t_2_*:


$$T=t_{2}-t_{1}$$

The actual rate of fiber biomass accumulation *V_t_* (g m^−2^ d^−1^) could be expressed as follows:


$$V_{t}=\frac{Y_{2}-Y_{1}}{t_{2}-t_{1}}$$

The time at which the maximum rate of increase in the fiber biomass occurred was *t_0_*:


$$t_{0}=-\frac{lna}{b}$$

When t=*t_0_*, the maximum rate of fiber biomass accumulation was *V_m_* (g m^−2^ d^−1^), and the theoretical maximum value *Y_m_* (g boll^-1^) could be written as follows:


$$V_{m}=-\frac{bK}{4}$$

$$Y_{m}=\frac{\text{K}}{1+{\text{ae}}^{{\text{bt}}_{0}}}$$

#### 2.3.4 Seed cotton and lint yield

During the harvest period, four representative small plot sampling points were randomly selected from each treatment, and 1–4 fruiting branches were divided into lower fruiting branches. Cotton samples from small plot sampling points with 5 or more upper fruiting branches were uniformly dried in air and weighed to calculate the seed cotton yield (kg ha^-1^). After ginning, the lint yield (kg ha^-1^) was measured, and the percentage of lint (%) was calculated.

Irrigation water use efficiency is calculated as follows (Irmak et al., 2020):


$$\text{IWUE}=\frac{\text{SCY}}{\text{I}}$$

where IWUE is the irrigation water use efficiency(kg m^3^), SCY is the seed cotton yield(kg ha^-1^);and I is the total irrigation amount during the entire growth period of drip-irrigated cotton, i.e., the irrigation quota(m^3^ ha^-1^)。

### 2.4 Statistical methods

All data were tested for normality (Shapiro–Wilk) and homogeneity of variance (Levene’s test) prior to analysis. The experiment followed a split-plot design with irrigation regime as the main plot and potassium level as the subplot, replicated three times per year. A combined ANOVA across 2021–2023 was performed using a linear mixed model: irrigation, potassium and their interactions were fixed effects; replication (nested within year) and the replication × irrigation term (main-plot error) were random effects. For traits measured at multiple boll ages (20–50 DPA), boll age and its interactions were added as fixed terms. Treatment means were compared by Duncan’s multiple range test at P < 0.05. All analyses were conducted in IBM SPSS Statistics v26.0. Pearson correlation analysis was used to assess pairwise relationships among physiological traits. Mantel tests were employed to evaluate the multivariate associations between each of the three agronomic indicators (lint yield, seed cotton yield, and IWUE) and the full set of physiological traits. Euclidean distance matrices were computed for each variable group after z-score standardization (mean = 0, SD = 1). Significance was determined by 9999 permutations. Principal component analysis (PCA) was performed to visualize overall treatment separation and trait relationships. The PCA dataset comprised 36 samples (6 treatments × 2 years × 3 replicates) and 14 variables (SS, SPS, β-1,3-glucanase, NR, GS, sucrose, β-1,3-glucan, free amino acids, soluble protein, S/P ratio, cellulose, fiber biomass, lint percentage, and lint yield). All variables were z-score standardized prior to PCA, and 95% confidence ellipses were drawn for each treatment group. Mantel tests and PCA were conducted in R (v. 4.3.1) using the “vegan” “FactoMineR” and “factoextra” packages. Figures were created using Origin 21.0.

## 3 Results

### 3.1 Water content of plant and cotton boll tissues

In 2021, the cotton boll water content in the I_500_ treatment group was significantly greater (by 2.8%) than that in the I_425_ treatment group during certain growth stages, whereas no significant differences were observed between the two irrigation regimes in 2022–2023 (**Figure 3**). With the same drip irrigation amount, the K_2_ and K_3_ treatments resulted in higher boll water contents than the K_1_ treatment did, with significant differences occurring at the late-full-boll stage. Compared with I_425_K_1_, I_500_K_3_ significantly increased the boll water content across multiple growth stages: by 1.2–8.3% across growth stages in all three years.

The results of the plant water content analysis indicated that compared with the I_425_ treatment (**Figure 3**), the I_500_ treatment resulted in significantly greater values throughout the growing season from 2021–2022 and during the early growth stages from full budding to full flowering in 2023; however, no significant difference was detected between them during the late growth stages (full boll to boll opening) in 2023. At the same drip irrigation level, compared with the K_1_ treatment, both the K_2_ and K_3_ treatments resulted in higher plant water contents, with significant differences being observed during the late growth period (full boll to boll opening) in 2021–2022 or from full budding to boll opening in 2023. No significant differences were found between I_425_ and I_500_ under the K_2_ or K_3_ conditions. Compared with the I_500_K_3_ treatment, the I_425_K_1_ treatment resulted in a significant decrease (1.1–2.2%), whereas no significant difference was detected between I_500_K_1_ and I_425_K_1_.

**Figure 3:**
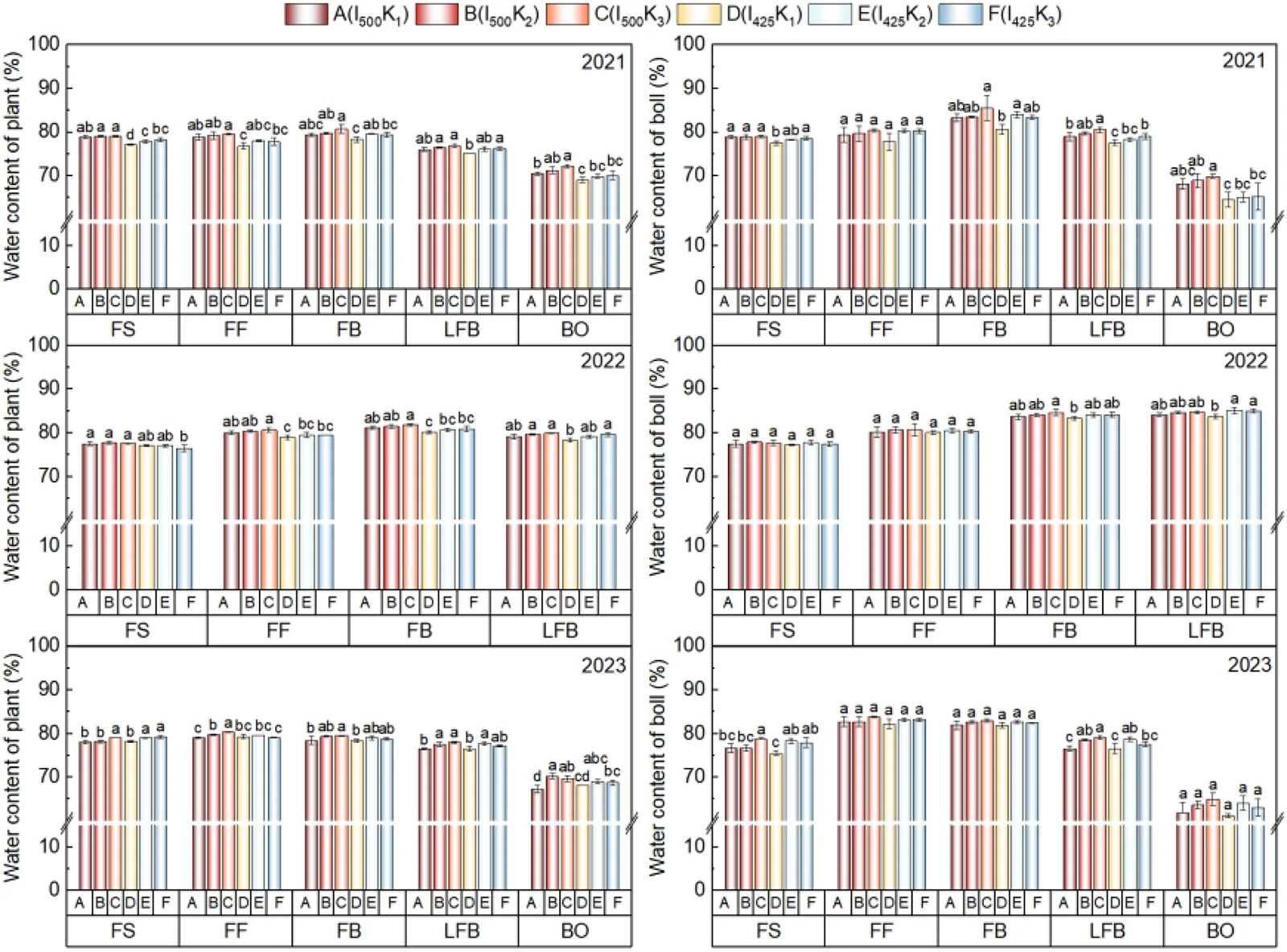
Effects of drip irrigation pattern and potassium application on the water contents of different organs of cotton (2021–2023). FS, full-squaring stage; FF, full-flowering stage; FB, full-boll stage; LFB, late-full-boll stage; and BO, boll-opening stage. Different lowercase letters within the same growth stage indicate significant differences among treatments at P < 0.05 according to Duncan’s multiple range test (n = 3).

### 3.2 Key enzymes in carbon metabolism

Drip irrigation significantly affected the activity of sucrose synthase (SS) in 30–40-day-old bolls in 2021 and in 40–50-day-old bolls in 2023 (**Table 2**). The SS activity under limited drip irrigation (I_425_) was 4.6% greater than that under conventional drip irrigation (I_500_) (except for a boll age of 20 days in 2021). The potassium application rate had a significant effect on the SS activity: Across both years, K_2_ and K_3_ significantly increased SS activity relative to K_1_ (by 7.1–10.3%), with no significant difference between the K_2_ and K_3_. The interaction effect between drip irrigation and potassium had a significant effect on the SS at 40–50 days in 2021 and 50 days in 2023. In 2021, compared with the other treatment groups, the I_425_K_2_ treatment resulted in the greatest SS, which significantly increased by 11.1%–21.2% at 40–50 days of age. The I_425_K_1_ treatment resulted in the lowest amount of SS. In 2023, I_425_K_2_ and I_425_K_3_ similarly exhibited the highest SS activity, and neither differed significantly from I_500_K_2_.

**Table 2:** Effects of the irrigation regime and potassium fertilization on the carbon–nitrogen ratio (2021–2023).

Year	2021				2023			
Treatment	Boll age 20 d	Boll age 30 d	Boll age 40 d	Boll age 50 d	Boll age 20 d	Boll age 30 d	Boll age 40 d	Boll age 50 d
I_500_K_1_	2.46a	1.29a	1.74ab	1.39ab	2.3a	1.12a	1.99ab	0.89ab
I_500_K_2_	2.36a	1.23a	1.69b	1.29b	2.27a	1.11a	1.83bc	0.85ab
I_500_K_3_	2.35a	1.25a	1.67b	1.37ab	2.29a	1.11a	1.79c	0.81b
I_425_K_1_	2.37a	1.21a	1.87a	1.53a	2.31a	1.13a	2.03a	0.92a
I_425_K_2_	2.46a	1.31a	1.66b	1.27b	2.19a	1.2a	1.69c	0.81b
I_425_K_3_	2.38a	1.26a	1.68b	1.34b	2.25a	1.13a	1.84bc	0.85ab
I_500_	2.39a	1.26a	1.7a	1.35a	2.29a	1.11a	1.87a	0.85a
I_425_	2.4a	1.26a	1.74a	1.38a	2.25a	1.15a	1.86a	0.86a
K_1_	2.41a	1.25a	1.81a	1.46a	2.31a	1.13a	2.01a	0.91a
K_2_	2.41a	1.27a	1.67b	1.28b	2.23a	1.15a	1.76b	0.83b
K_3_	2.36a	1.25a	1.68b	1.35ab	2.27a	1.12a	1.82b	0.83b
I	ns	ns	ns	Ns	ns	ns	ns	ns
K	ns	ns	*	*	ns	ns	**	*
I*K	ns	ns	ns	Ns	ns	ns	ns	ns

S/P ratio, sucrose-to-soluble-protein ratio; I, irrigation main effect; K, potassium main effect; I×K, irrigation–potassium interaction; ns, not significant; *P < 0.05; **P < 0.01. Values with different letters within the same column and year differ significantly at P < 0.05 (Duncan’ s test, n = 3).

Drip irrigation significantly affected sucrose phosphate synthase (SPS) activity in 20–40-day-old bolls in 2021 and 20-day-old bolls in 2023. The SPS of limited drip irrigation (I_425_) was 5.8% greater than that of conventional drip irrigation (I_500_) (except for that at a boll age of 20 days in 2021). The potassium application rate had a significant effect on the SPS activity (except for the 30-day boll age in 2023), and the K_2_ (medium) and K_3_ (high) treatment groups were significantly greater than the activity in the K_1_ (low) treatment group by 12.1% and 12.0%, respectively. The SPS levels in the K_2_ (medium) and K_3_ (high) treatment groups were not significantly different. The interaction effect between only drip irrigation and potassium was significant for the SPS level at 20–40 days in 2023. In both years, I_425_K_2_ consistently yielded the highest SPS activity, with increases of 8.2%–21.4% relative to I_500_K_1_, I_500_K_3_ and I_425_K_1_. Furthermore, the SPS levels in the I_425_K_2_ and I_425_K_3_ treatment groups in 2021 and 2023 were not significantly different.

Drip irrigation significantly affected the activity of β-1,3-glucanase, and the β-1,3-glucanase activity under limited drip irrigation conditions (I_425_) was 6.1% greater than that under conventional drip irrigation conditions (I_500_), except for the 20-day boll age in 2021. The potassium application rate significantly affected the activity of β-1,3-glucanase. Compared with the level in the K_1_ (low) treatment group, the β-1,3-glucanase activity in the K_2_ (medium) and K_3_ (high) treatment groups significantly increased by 20.2% and 18.7%, respectively. The β-1,3-glucanase activity in the K_2_ (medium) and K_3_ (high) treatment groups in 2023 were not significantly different. The interaction effect between the drip irrigation rate and potassium concentration significantly affected the β-1,3-glucanase activity of 20–40-day-old bolls in 2021 and 20-day-old bolls in 2023. At a boll age of 30–50 days in 2021, the β-1,3-glucanase activity in the I_425_K_2_ treatment group increased by 36.2%, 11.3%, 11.1%, 25.9%, and 7.5% compared with that in the I_500_K_1_, I_500_K_2_, I_500_K_3_, I_425_K_1_, and I_425_K_3_ treatment groups, respectively. Across both years, I_425_K_2_ consistently showed the highest β-1,3-glucanase activity, with increases of 5.8%–36.2% over the other five treatments; the largest gains were relative to I_500_K_1_ and I_425_K_1_, while I_425_K_2_ and I_500_K_3_ were statistically comparable at 50 days in 2021.

### 3.3 Key enzymes in fiber nitrogen metabolism

As shown in **Figure 4**, increasing the amount of potassium fertilizer applied in the two different drip irrigation modes increased the activities of NR and GS in the cotton fibers. Under conventional drip irrigation conditions, NR increases with increasing potassium application rate. At a boll age of 20–50 days, compared with that under low-potassium conditions, the NR activity levels under potassium and high-potassium conditions increased by 3.4–29.0% and 3.8–32.0%, respectively, whereas the GS activity levels increased by 8.6–15.9% and 14.1–31.0%, respectively. Under limited-potassium conditions, the NR activity tended to first increase but then decrease with increasing potassium application rate. Compared with that under low-potassium conditions, the NR activity levels under medium-potassium conditions and high-potassium conditions increased by 9.7–33.2% and 7.3–29.9%, respectively, at ages 20–50 days, and the GS activity levels increased by 7.3–30.1% and 4.5–28.6%, respectively. These findings indicated that increasing the potassium fertilizer level under both drip irrigation rates could increase the activities of key enzymes involved in cotton fiber nitrogen metabolism; moreover, the moderate-potassium treatment performed the best under limited drip irrigation conditions.

**Figure 4:**
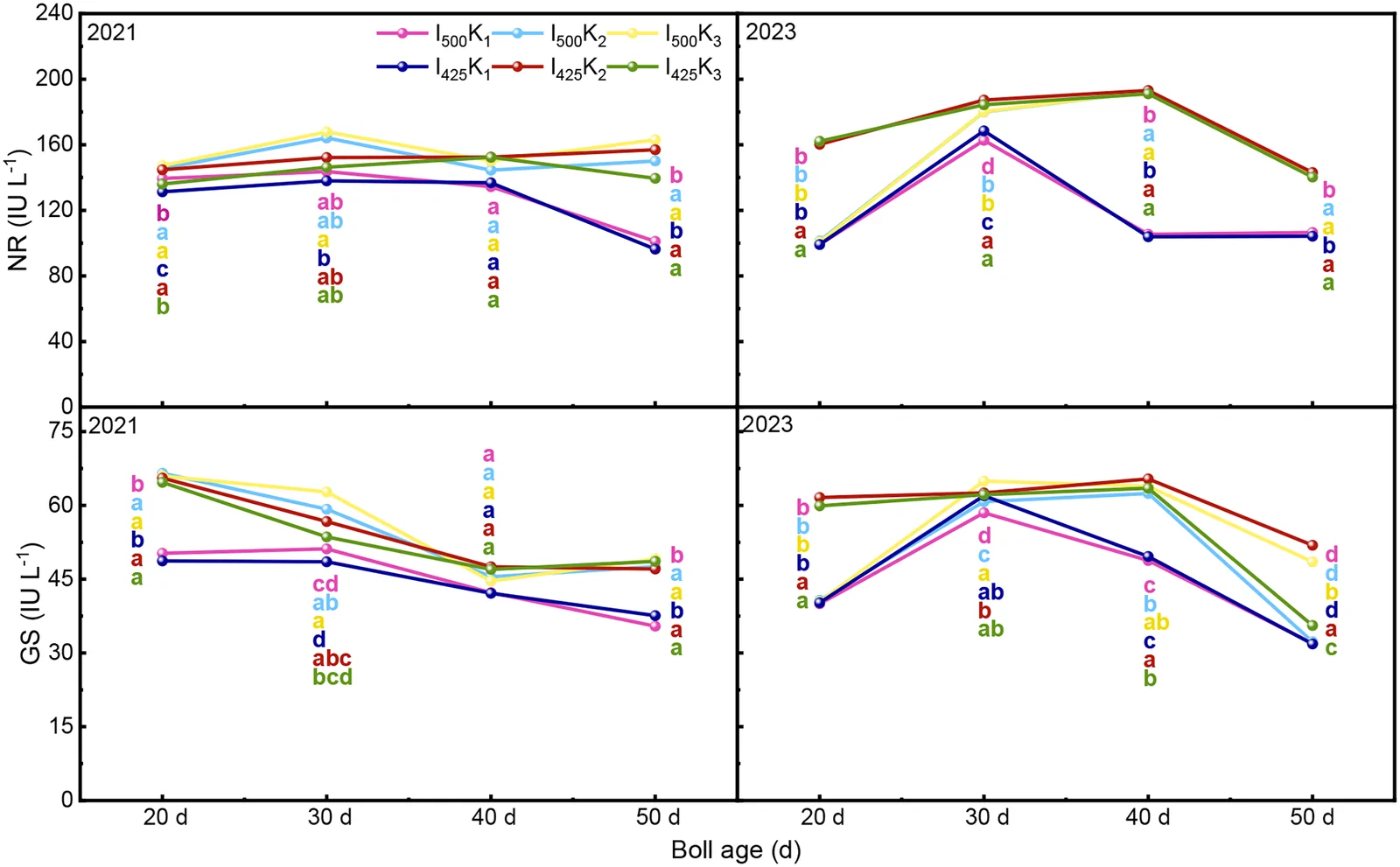
Effects of the irrigation regime and potassium fertilization on nitrate reductase activity and glutamine synthetase activity in cotton fibers (2021 and 2023). Different lowercase letters within the same boll age indicate significant differences among treatments at P < 0.05 according to Duncan’s multiple range test (n = 3). NR, Nitrate reductase; GS, Glutamine synthetase.

### 3.4 Carbon metabolism-related substances

The drip irrigation mode had no significant effect on the sucrose content (**Figure 5**). The potassium application rate had a significant effect on the sucrose content of bolls aged 20–30 d in 2021 and 30 d in 2023. At 20–30 d of boll age, compared with that in the K_1_ treatment group, the sucrose contents of bolls subjected to the K_2_ and K_3_ treatments significantly increased by 4.9% and 4.6%, respectively. At 40–50 days of boll age, the sucrose content tended to increase in the order of K_1_>K_2_ and K_3_, but the difference was not significant. The interaction effect between drip irrigation and potassium application had a significant effect on the sucrose content at 30 days of boll age in 2021. Statistical analysis revealed that the sucrose content did not differ significantly between the I_425_K_2_ and I_425_K_3_ treatment groups. The sucrose contents in the I_425_K_2_ and I_425_K_3_ treatment groups at 20–30 days of boll age increased by average values of 5.5%, 3.1%, 3.4%, and 7.9% compared with the values the I_500_K_1_, I_500_K_2_, I_500_K_3_, and I_425_K_1_ treatment groups. The sucrose contents increased by 2.6%, 3.4%, 5.0%, 6.9%, and 5.3% in the I_425_K_1_ treatment group at 40–50 days of age compared with the values in the I_500_K_1_, I_500_K_2_, I_500_K_3_, I_425_K_2_, and I_425_K_3_ treatment groups.

**Figure 5:**
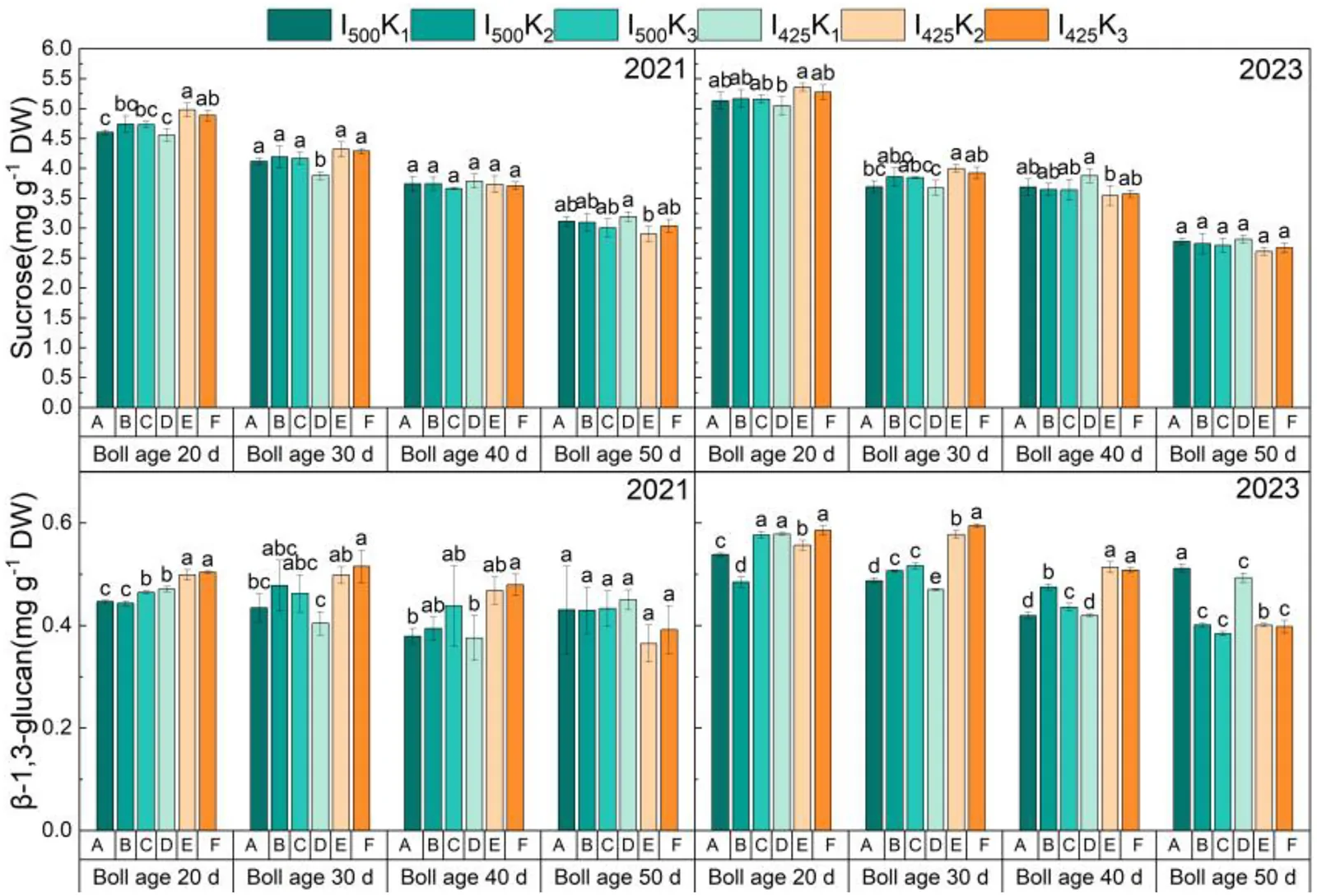
Effects of irrigation regime and potassium fertilization on sucrose and β-1,3-glucan contents in cotton fibers (2021 and 2023). Different lowercase letters within the same boll age indicate significant differences among treatments at P < 0.05 according to Duncan’s multiple range test (n = 3).

The drip irrigation mode significantly affected the contents of β-1,3-glucan in 20-day-old bolls in 2021 and in 20–40-day-old bolls in 2023 (**Figure 5**). The content of β-1,3-glucan under limited drip irrigation conditions (I_425_) at 20–40 days of boll age was 7.6% greater than that under conventional drip irrigation conditions (I_500_). The potassium application rate had a significant effect on the content of β-1,3-glucan (except at 50 days of boll age in 2021). At 20–40 days of age, the contents of β-1,3-glucan in the K_2_ (medium) and K_3_ (high) treatment groups significantly increased by 8.8% and 12.2%, respectively, compared with the value in the K_1_ (low) treatment group. At 50 days of boll age, the content of β-1,3-glucan tended to increase in the order of K_1_>K_2_>K_3_. The interaction effect between the drip irrigation rate and the potassium application rate significantly affected the content of β-1,3-glucan at 20 d of boll age in 2021 and 20–50 d of boll age in 2023. The content of β-1,3-glucan increased by 15.1% and 17.9%, by 11.9% and 14.5%, by 7.5% and 10.1%, and by 14.7% and 17.4% in the I_425_K_2_ and I_425_K_3_ treatments at a boll age of 20–40 days compared with the values in the I_500_K_1_, I_500_K_2_, I_500_K_3_, and I_425_K_1_ treatment groups, respectively. The content of β-1,3-glucan increased by 13.4%, 15.2%, 23.0%, and 19.4% in the I_500_K_1_ treatment group at 50 days of boll age compared with the values in the I_500_K_2_, I_500_K_3_, I_425_K_2_, and I_425_K_3_ treatment groups, respectively. The β-1,3-glucan contents in the I_500_K_1_ and I_425_K_1_ treatment groups were not significantly different.

### 3.5 Substances related to nitrogen metabolism

As shown in **Figure 6**, the changes in the free amino acid (FAA) content in cotton fibers at the two drip irrigation rates differed from those at the different potassium application rates: Under I_500_, the application of potassium fertilizer led to a decrease in FAA content, with potassium and high-potassium FAA contents decreasing by 2.6%–6.1% and 1.9%–4.4%, respectively, compared with the low-potassium FAA content in 20–50-day-old bolls. Under I_425_, the application of potassium fertilizer significantly increased the fiber FAA content of cotton with a boll age of 20–50 days. Compared with those under low-potassium conditions, the FAA contents under medium- and high-potassium conditions increased by 1.2–7.0% and 2.9–7.3%, respectively.

**Figure 6:**
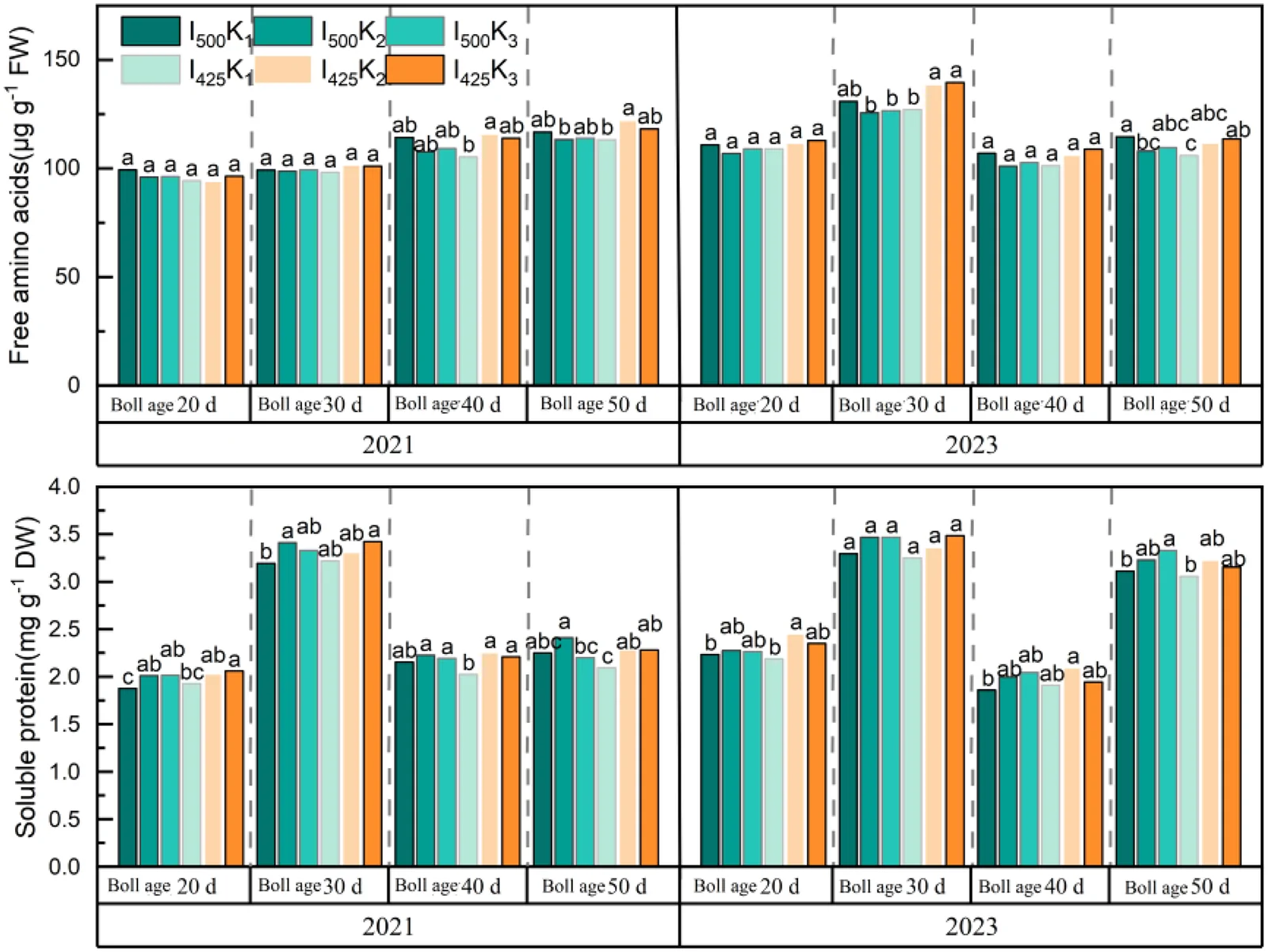
Effects of the irrigation regime and potassium fertilization on the contents of free amino acids and soluble protein in cotton fibers (2021 and 2023). Different lowercase letters within the same boll age indicate significant differences among treatments at P < 0.05 according to Duncan’s multiple range test (n = 3).

As shown in **Figure 6**, compared with that in the low-potassium treatment group, the soluble protein contents in the cotton fibers in the medium-potassium and high-potassium treatment groups increased under the two different drip irrigation rates by 3.0%–9.5% and 5.2%–6.8% under limited drip irrigation conditions and by 4.2%–5.7% and 4.0%–5.3% under conventional drip irrigation conditions, respectively. This finding indicated that increasing levels of potassium could increase the soluble protein contents of cotton fibers regardless of sufficient water supply and that the increase was greater under limited drip irrigation.

### 3.6 Cotton fiber sucrose-to-soluble-protein ratio

The S/P ratio of the cotton fibers remained stable among the drip irrigation modes, with no significant differences being observed. There were no significant differences in the S/P ratio of the cotton fibers in the 20–30-day boll age treatment groups with the potassium application rates; however, compared with the K_2_ (medium) and K_3_ (high) treatments, the K_1_ (low) treatment significantly increased the S/P ratio at a boll age of 40–50 days. Under conventional drip irrigation conditions, the K_1_ (low) treatment significantly increased the ratio by 5.5%–5.8% and by 5.2%–7.0% compared with the K_2_ (medium) and K_3_ (high) treatments, respectively. Under limited drip irrigation conditions, the K_1_ (low) treatment significantly increased the ratios by 12.0%–16.7% and 7.6%–9.4% compared with the K_2_ (medium) and K_3_ (high) treatments. No significant interactive effect was observed in terms of the drip irrigation regime and the potassium fertilization level on the S/P ratios of cotton fibers at 20–30 days of boll age. The S/P ratios of the cotton fibers in the I_425_K_1_ group significantly increased by 9.7%–11.6%, 11.2%–11.3%, 14.0%–14.5%, and 9.8%–10.0% compared with those in the I_500_K_2_, I_500_K_3_, I_425_K_2_, and I_425_K_3_ treatment groups, respectively. There were no significant differences in the S/P ratios of cotton fibers treated with I_425_K_1_ and I_500_K_1_.

### 3.7 Cellulose content

Compared with I_500_, potassium significantly increased the cellulose content by 3.9–8.4% under limited drip irrigation conditions. The use of potassium fertilizer increased the cellulose content under both drip irrigation modes. Under conventional drip irrigation conditions, the K_2_ (medium) and K_3_ (high) treatments increased the contents by 3.4–13.1% and 3.4–13.8%, respectively, compared with that of the K_1_ (low) treatment, and the difference was significant by 2023. Under limited drip irrigation conditions, the cellulose content tended to first increase but then decrease with increasing potassium application rate, with those in the K_2_ (medium) and K_3_ (high) treatment groups increasing by 17.8–24.6% and 14.3%–19.9%, respectively, compared with those in the K_1_ treatment group. The cellulose contents increased by 22.7%, 12.1%, 12.7%, 26.3%, and 5.3% in the I_425_K_2_ treatment group at a boll age of 20–50 days compared with the I_500_K_1_, I_500_K_2_, I_500_K_3_, I_425_K_1_, and I_425_K_3_ treatment groups. Statistical analysis revealed no significant difference in cellulose content between the I_425_K_2_ and I_425_K_3_ treatment groups at a boll age of 40–50 days in 2023. The cellulose content was the lowest in I_425_K_1_. A comparison between the I_500_K_1_ and I_425_K_1_ treatments revealed no significant difference in their cellulose content.

### 3.8 Fiber biomass

There was a significant difference in the fiber biomass between the drip irrigation mode and the potassium application rate. Compared with I_500_, I_425_ increased the dry matter content of the fibers by 11.8%. The biomass of cotton fibers tended to first increase but then decrease with increasing potassium content (**Figure 7**). Compared with that of K_1_ (low), the upper-fiber biomass of K_3_ increased by 4.2% and 7.7% under I_500_ and I_425_, respectively. Compared with that of K_3_, the upper-fiber biomass of K_2_ increased by 4.8% and 7.4% under I_500_ and I_425_, respectively. Compared with those of K_1_ and K_3_, the lower fiber biomass of K_2_ increased by 16.5% and 16.9% and by 11.5% and 5.6%, respectively, under I_500_ and I_425_. These findings indicated that in both drip irrigation modes, an appropriate increase in potassium fertilizer (K_2_) application was beneficial for increasing the cotton boll fiber biomass.

**Figure 7:**
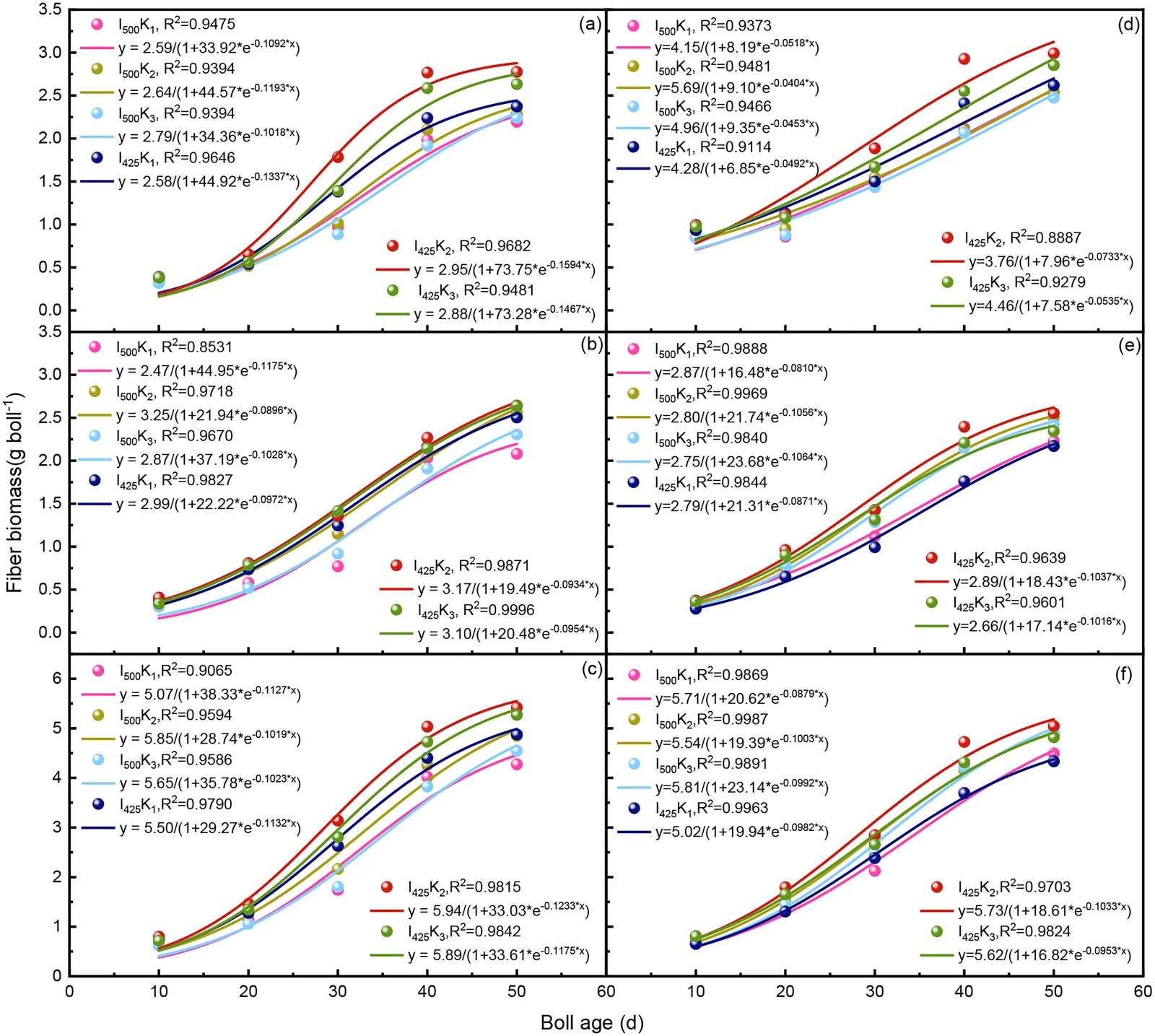
Logistic equation fitting for the fiber biomass of cotton plants (2021 and 2023). **(a)** 2021-UFB; **(b)** 2023-UFB; **(c)** 2021-LFB; **(d)** 2023-LFB; **(e)** 2021-T; and **(f)** 2023-T.

The accumulation levels of fiber biomass in the upper fruiting branches (UFBs), lower fruiting branches (LFBs), and entirety (T) of the cotton plants were fitted using logistic growth curves (**Figure 7**). The goodness-of-fit parameters indicated a strong model fit, and the fiber biomass accumulation level across all the plant components consistently followed the characteristic sigmoidal growth pattern, typified by initial slow, subsequent rapid, and final slow growth phases.

Upper fruiting branches (UFBs) (**Table 3**): The I_425_ treatment significantly advanced the rapid accumulation phase. For instance, at the K_2_ level in 2021, *t_0_*, *t_1_*, and *t_2_* advanced by 4.86, 2.08, and 7.64 days, respectively. However, this level shortened the duration of rapid accumulation (T) and reduced the theoretical maximum biomass (*Y_m_*). The effect of potassium was modulated by the irrigation mode. For the I_500_ treatment, higher potassium levels increased the yield potential (e.g., the *Y_m_* of I_500_K_2_ reached 2.85 g boll^-1^ in 2023). In contrast, under I_425_K_2_, optimal performance was demonstrated (e.g., the *Y_m_* of I_425_K_2_ was 1.48 g boll^-1^ in 2021).

**Table 3:** Logistic equations to fit parameters characterizing fiber biomass accumulation (2021 and 2023).

Fruiting branch	Treatment	2021							2023						
		*t_0_*	*t_1_*	*t_2_*	*T*	*V_t_*	*V_m_*	*Y_m_*	*t_0_*	*t_1_*	*t_2_*	*T*	*V_t_*	*V_m_*	*Y_m_*
UFB	I_500_K_1_	32.27	20.21	44.33	24.12	0.06	0.07	1.3	40.6	15.18	66.01	50.84	0.05	0.05	2.07
	I_500_K_2_	31.83	20.79	42.87	22.08	0.07	0.08	1.32	54.69	22.07	87.31	65.24	0.05	0.06	2.85
	I_500_K_3_	34.74	21.81	47.68	25.87	0.06	0.07	1.39	49.38	20.29	78.47	58.18	0.05	0.06	2.48
	I_425_K_1_	28.46	18.61	38.32	19.70	0.08	0.09	1.29	39.13	12.35	65.92	53.57	0.05	0.05	2.14
	I_425_K_2_	26.97	18.71	35.23	16.52	0.10	0.12	1.48	28.31	10.34	46.28	35.94	0.06	0.07	1.88
	I_425_K_3_	29.27	20.29	38.25	17.95	0.09	0.11	1.44	37.86	13.25	62.48	49.22	0.05	0.06	2.23
LFB	I_500_K_1_	32.40	21.19	43.61	22.43	0.06	0.07	1.24	34.61	18.35	50.88	32.53	0.05	0.06	1.44
	I_500_K_2_	34.48	19.78	49.18	29.41	0.06	0.07	1.63	29.16	16.69	41.64	24.95	0.06	0.07	1.40
	I_500_K_3_	35.18	22.36	47.99	25.62	0.06	0.07	1.43	29.76	17.37	42.14	24.76	0.06	0.07	1.37
	I_425_K_1_	31.89	18.34	45.43	27.09	0.06	0.07	1.49	35.11	19.99	50.22	30.23	0.05	0.06	1.40
	I_425_K_2_	31.79	17.69	45.89	28.19	0.06	0.07	1.59	28.10	15.40	40.81	25.40	0.07	0.07	1.45
	I_425_K_3_	31.64	17.84	45.44	27.6	0.06	0.07	1.55	27.96	15.00	40.93	25.92	0.06	0.07	1.33
T	I_500_K_1_	32.35	20.66	44.03	23.37	0.13	0.14	2.54	34.43	19.45	49.41	29.96	0.11	0.13	2.85
	I_500_K_2_	32.97	20.04	45.9	25.86	0.13	0.15	2.93	29.57	16.43	42.70	26.27	0.12	0.14	2.77
	I_500_K_3_	34.96	22.09	47.82	25.74	0.13	0.14	2.83	31.66	18.39	44.94	26.55	0.13	0.14	2.91
	I_425_K_1_	29.84	18.20	41.48	23.28	0.14	0.16	2.75	30.47	17.06	43.88	26.82	0.11	0.12	2.51
	I_425_K_2_	28.37	17.69	39.06	21.37	0.16	0.18	2.97	28.30	15.55	41.04	25.49	0.13	0.15	2.87
	I_425_K_3_	29.93	18.71	41.14	22.43	0.15	0.17	2.94	29.62	15.80	43.44	27.64	0.12	0.13	2.81

*Y_m_*, Theoretical maximum (g boll^−1^); *t_0_*, Maximum rate emergence time (d); *t_1_*, Rapid onset time of fiber biomass accumulation (d); *t_2_*, Rapid end time of accumulation (d); T, Rapid accumulation duration (d); *V_t_*, Actual rate (g m^−2^ d^−1^); and *V_m_*, Maximum rate (g m^−2^ d^−1^).

Lower fruiting branches (LFBs) (**Table 3**): This plant part exhibited greater sensitivity to water stress, with an average 11.0% reduction in *Y_m_* under limited irrigation conditions (I_425_) in 2023. The effect of potassium varied considerably across years. In 2021, the K_2_ level extended the duration of rapid accumulation and increased *Y_m_* by up to 20.9%. Conversely, in 2023 under conventional irrigation conditions (I_500_), the low-potassium level (K_1_) unexpectedly yielded the best performance (*Y_m_* of 2.23 g boll^-1^).

Whole plant level (**Table 3**): Although water stress promoted an earlier onset of the rapid accumulation phase, it significantly suppressed the fiber yield, as evidenced by the decreased *Y_m_* in all I_425_ treatments in 2023. Notable water–potassium interactions were observed. Under conventional irrigation conditions (I_500_), medium to high potassium levels increased the yield (e.g., the *Y_m_* of I_500_K_2_ was 2.93 g boll^-1^ in 2021). Under limited irrigation conditions (I_425_), the medium-potassium level (K_2_) achieved the best compensatory effect by markedly increasing the maximum accumulation rate (the *V_m_* of I_425_K_2_ reached 0.18 g m^-2^ d^-1^ in 2021), resulting in a *Y_m_* of 2.97 g boll^-1^.

In summary, cotton fiber accumulation demonstrated distinct location-specific characteristics and significant water–potassium interactions. The combination of a medium-potassium level with appropriate irrigation represented the optimal cultivation management strategy, facilitating synergistic yield increases across different plant parts.

### 3.9 Lint yield and Lint percentage

Irrigation and potassium jointly shaped lint yield (P<0.01, 2022–2023) (**Table 4**), while only irrigation altered lower-branch yield in 2021. Averaged across years, limited irrigation (I_425_) boosted upper lint yield by 19.7% (2022–2023) and lower yield by 8.0% versus full irrigation (I_500_). Upper and lower yields rose then fell with potassium rate: medium K (K_2_) outperformed high K (K_3_) and low K (K_1_). Total yield under I_425_ exceeded I_500_ by 8.1%; I_425_K_2_ achieved peak yield, followed by I_425_K_3_. The I_500_K_1_ treatment yielded 18.3% less than all other combinations.

I_425_ consistently elevated upper, lower and whole-plant lint percentage (**Table 4**) by 0.4–1.4%, 2.4–4.9% and 1.4–3.5% respectively relative to I_500_, with larger gains on lower branches. Potassium improved lint fraction, particularly K_2_. I_425_K_2_ produced the highest lint percentage across canopy layers, comparable to I_425_K_3_. Low-K treatments (I_500_K_1_, I_425_K_1_) had markedly reduced lint percentage, proving water shortage coupled with K deficiency impairs fiber assimilate allocation.

**Table 4:** Effects of the drip irrigation mode and potassium application rate on cotton yield and lint percentage in different fruiting branch parts (2021–2023).

Year	Treatment	Lint yield (kg ha^-1^)			Lint percentage (%)		
		UFB	LFB	T	UFB	LFB	T
2021	I_500_K_1_	1413.3a	1744.6bc	3160.3b	43.2ab	42.6c	42.9b
	I_500_K_2_	1374.3a	1793.4abc	3166.3b	43.2ab	43.4bc	43.3b
	I_500_K_3_	1335.3a	1676.4c	3009.6c	42.3b	43.0c	42.7b
	I_425_K_1_	1218.3a	1820.5ab	3028.7bc	43.2ab	44.7ab	43.9ab
	I_425_K_2_	1432.7a	1914.8a	3346.7a	44.9a	45.1a	45.1a
	I_425_K_3_	1286.5a	1869ab	3141.8bc	42.2b	44.8ab	43.5ab
	I	Ns	**	ns	ns	**	**
	K	Ns	ns	**	*	ns	ns
	I * K	Ns	ns	**	ns	ns	ns
2022	I_500_K_1_	810.9d	1809.6d	2648.5e	41.4d	40.6c	41.0d
	I_500_K_2_	1002.4c	2238.4b	3252.6c	44.5ab	42.6b	43.6bc
	I_500_K_3_	1193.6b	2326.0b	3528.7b	43.8bc	42.5b	43.2c
	I_425_K_1_	930.3cd	2015.9c	2933.3d	42.9c	43.7ab	43.3bc
	I_425_K_2_	1488.3a	2579.4a	4092.0a	45.1a	44.9a	44.9a
	I_425_K_3_	1375.9a	2484.4a	3892.2a	44.6ab	43.7ab	44.1ab
	I	**	**	**	**	**	**
	K	**	**	**	**	**	**
	I * K	**	ns	**	ns	ns	ns
2023	I_500_K_1_	744.49c	2047.37c	2800.32e	42.7b	42.1b	42.4e
	I_500_K_2_	826.79bc	2172.97b	3025.96cd	43.8a	42.2b	42.9c
	I_500_K_3_	878.17ab	2212.85b	3114.18bc	43.3ab	41.9b	42.6b
	I_425_K_1_	838.65bc	2081.09c	2924.72d	42.6b	42.3b	42.5d
	I_425_K_2_	973.68a	2439.19a	3404.68a	43.9a	44.4a	44.1a
	I_425_K_3_	922.61ab	2250.29b	3193.87b	43.8a	42.5b	43.2a
	I	**	**	**	ns	**	**
	K	**	**	**	**	**	**
	I * K	Ns	**	**	ns	**	*

Each value represents the mean (n=3); values assigned distinct letters are significantly different at P < 0.05. UFB, LFB and T denote the upper fruiting branch, lower fruiting branch and total cotton plant, respectively; *There is a significant difference at the P=0.05 level; **There is a significant difference at the P=0.01 level.

### 3.10 Seed cotton yield and IWUE

The response of cotton seed yield to drip irrigation was potassium dependent. Under low potassium conditions (K_1_), conventional irrigation (I_500_) outperformed limited irrigation (I_425_) in 2021 only. Under medium or high potassium (K_2_ or K_3_, respectively) levels, limited irrigation (I_425_) significantly increased upper and lower seed cotton yield in 2022, with gains of 31.7% and 11.8% (upper) and 17.9% and 7.3% (lower), respectively, whereas no yield differences were observed between irrigation regimes in 2023. Potassium effects varied with irrigation. Under conventional irrigation conditions (I_500_), the seed cotton yield increased proportionally with the potassium rate from 2022–2023, with K_2_ and K_3_ increasing the total yield by 9.3% and 15.6%, respectively. Under limited irrigation conditions (I_425_), seed cotton yield first increased but then decreased with increasing potassium; the greatest gains occurred for K_2_ in 2022 for the upper yield (34.2% increase) and for K_3_ for the lower yield. The highest total seed cotton yield was achieved with I_425_K_2_ or I_425_K_3_.

The irrigation water use efficiency (IWUE) was significantly greater under I_2_ than under I_500_ across all potassium levels (**Table 5**), with increases of 18.7%, 26.6%, and 18.9% for K_1_, K_2_, and K_3_, respectively. Under I_500_, the IWUE increased with increasing potassium concentration (16.1% and 15.6% for K_2_ and K_3_, respectively, compared with K_1_). Under I_425_, IWUE peaked at K_2_, with K_2_ and K_3_ increasing the IWUE by 16.0% and 15.7%, respectively, relative to K_1_. Thus, potassium application enhanced the IWUE under both irrigation regimes, with medium potassium under limited irrigation being particularly effective.

**Table 5:** Effects of the drip irrigation mode and potassium application rate on the seed cotton yield and IWUE of different fruiting branch parts (2021–2023).

Year	Treatment	Seed cotton yield (kg ha^-1^)			IWUE (kg m^3^)
		UFB	LFB	T	
2021	I_500_K_1_	3274.85 ± 109.41a	4093.57 ± 47.75ab	7368.42 ± 156.55a	1.48 ± 0.031c
	I_500_K_2_	3187.13 ± 227.75a	4132.55 ± 49.7ab	7319.69 ± 180.77a	1.47 ± 0.036c
	I_500_K_3_	3157.89 ± 208.13a	3898.64 ± 107.65b	7056.53 ± 143.91ab	1.41 ± 0.029c
	I_425_K_1_	2816.76 ± 185.84a	4077.19 ± 54.25ab	6893.96 ± 183.55b	1.62 ± 0.043b
	I_425_K_2_	3187.13 ± 47.75a	4241.91 ± 106.06a	7429.04 ± 145.01a	1.75 ± 0.034a
	I_425_K_3_	3050.68 ± 137.84a	4168.62 ± 201.14a	7219.3 ± 170.58ab	1.7 ± 0.040ab
2022	I_500_K_1_	1961.17 ± 49.64c	4450.61 ± 181.48b	6460.39 ± 115.37d	1.29 ± 0.023d
	I_500_K_2_	2252.81 ± 130.55c	5250.65 ± 171.07b	7467.36 ± 235.25c	1.49 ± 0.047c
	I_500_K_3_	2724.24 ± 158.25b	5470.1 ± 146.88ab	8173.92 ± 157.76b	1.64 ± 0.032b
	I_425_K_1_	2170.81 ± 266.45c	4614.6 ± 107.41b	6776.65 ± 281.74d	1.60 ± 0.066bc
	I_425_K_2_	3297.66 ± 88.05a	5752.82 ± 158.51a	9094.98 ± 175.6a	2.14 ± 0.041a
	I_425_K_3_	3087.22 ± 189.79a	5684.56 ± 158.28a	8819 ± 238.88a	2.08 ± 0.056a
2023	I_500_K_1_	1744.64 ± 72.94b	4863.55 ± 70.22b	6608.19 ± 38.47c	1.32 ± 0.008e
	I_500_K_2_	1890.84 ± 117.77ab	5155.95 ± 83.84ab	7046.78 ± 143.25abc	1.41 ± 0.029d
	I_500_K_3_	2028.2 ± 86.79ab	5282.65 ± 113.59ab	7310.85 ± 189.08abc	1.46 ± 0.038d
	I_425_K_1_	1968.81 ± 121.73ab	4922.03 ± 56.85b	6890.84 ± 176.03bc	1.62 ± 0.041c
	I_425_K_2_	2222.22 ± 167.12a	5497.08 ± 63.17a	7719.30 ± 189.5a	1.82 ± 0.045a
	I_425_K_3_	2105.26 ± 109.34ab	5292.4 ± 97.1ab	7397.66 ± 46.6ab	1.74 ± 0.011b

Each value represents the mean (n=3); values assigned distinct letters are significantly different at P < 0.05. UFB, LFB and T denote the upper fruiting branch, lower fruiting branch and total cotton plant, respectively. IWUE represents the irrigation water use efficiency.

### 3.11 Correlations analysis

Pearson correlation showed close relationships among carbon/nitrogen metabolic enzymes, cell wall carbohydrates, fiber traits and boll water content (**Figure 8**. heatmap panel): Carbohydrate metabolic enzymes (SS, SPS, β-1,3-Gase) had extremely significant positive intercorrelations (P<0.001), and were positively associated with NR, GS, cellulose, fiber biomass and lint percentage (LP, P<0.001). Nitrogen metabolites (FAA, soluble protein) positively correlated with NR/GS, while S/P ratio showed strong negative correlations with FAA and soluble protein (P<0.001). BWC only weakly positively correlated with SS, SPS and β-1,3-Gase (0.001<P ≤ 0.01), with no significant link to GS.

**Figure 8:**
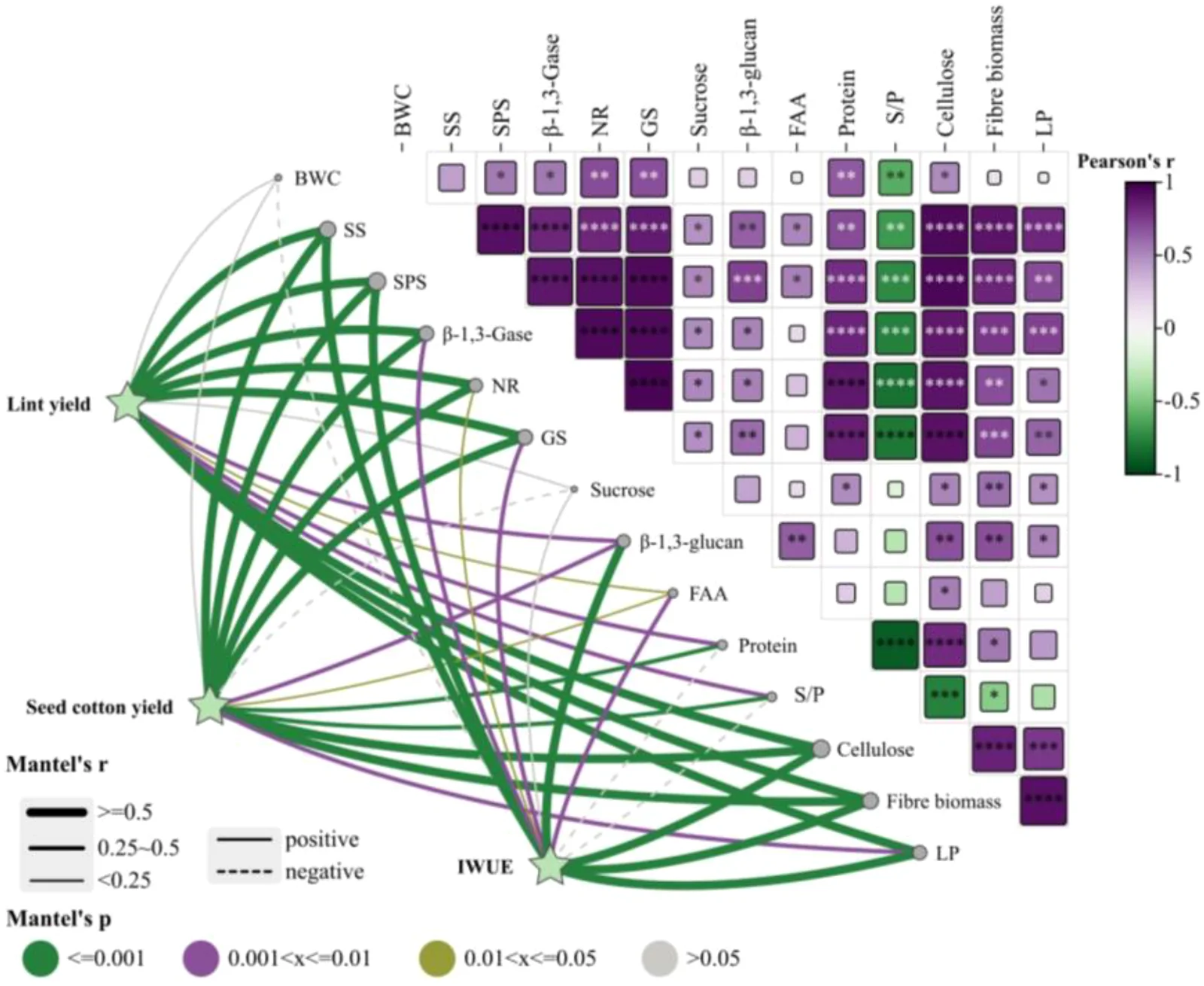
Pairwise pearson correlations and Mantel test associations among cotton physiological, yield and water use efficiency traits. The upper heatmap panel displays pairwise Pearson’s correlation coefficients between boll water status, carbon/nitrogen metabolic enzymes, soluble metabolites, cell wall polysaccharides and fiber-related indicators; purple blocks represent positive correlations, green blocks denote negative correlations, and asterisks indicate significance levels. The network panel illustrates Mantel test multivariate correlations of lint yield, seed cotton yield and integrated water use efficiency (IWUE) with all physiological indices. Line thickness corresponds to Mantel’s r magnitude, solid green lines represent positive correlations, dashed purple lines represent negative correlations. Line color indicates Mantel’s significance: green (P ≤ 0.001), purple (0.001 < P ≤ 0.01), yellow (0.01 < P ≤ 0.05), grey (P > 0.05). BWC, boll water content; SS, sucrose synthase; SPS, sucrose phosphate synthase; β-1,3-Gase, β-1,3-glucanase; NR, nitrate reductase; GS, glutamine synthetase; FAA, free amino acids; Protein, soluble protein; S/P, sucrose-to-soluble-protein ratio (sucrose on a DW basis divided by soluble protein on an FW basis); LP, lint percentage.

Mantel test quantified the magnitude and significance of multivariate associations between three core agronomic indicators (lint yield, seed cotton yield, IWUE) and the full set of physiological traits (**Figure 8**, network panel). Strong positive Mantel correlations (Mantel’s r ≥ 0.5, P ≤ 0.001) were observed between lint yield and SS, SPS, β-1,3-Gase, NR, cellulose, fiber biomass, and LP.; moderate positive correlations with GS and β-1,3-glucan. Weak positive correlations were found with sucrose, FAA, soluble protein (0.001<P ≤ 0.01), while a weak negative correlation occurred with S/P. No significant correlation was detected with BWC (P>0.05). Seed cotton yield shared identical correlation patterns with physiological indicators as lint yield: strong positive associations with carbon/nitrogen metabolic enzymes and fiber structural components, weak positive links with sugar, amino acid and soluble protein, weak negative correlation with S/P, and non-significant correlation with BWC. IWUE exhibited the broadest range of strong positive correlations (Mantel’s r≥0.5, P ≤ 0.001), covering SS, SPS, β-1,3-Gase, NR, GS, cellulose, fiber biomass and LP. It had moderate positive correlation with β-1,3-glucan, weak positive correlations with sucrose, FAA, soluble protein, weak negative correlation with S/P, and no significant correlation with BWC. Lint yield, seed cotton yield and IWUE were mutually strongly positively correlated (Mantel’s r≥0.5, P ≤ 0.001).

### 3.12 Principal component analysis

Principal component analysis (PCA) resolved 81.9% of total phenotypic variation across all physiological, yield and water-use traits via the first two principal axes, with PC1 explaining 71.5% and PC2 explaining 10.4% of cumulative variance (**Figure 11**); all irrigation-potassium treatment groups exhibited clear spatial separation along PC1 and PC2 with non-overlapping confidence ellipses, where samples under full irrigation (I_500_; red, blue, orange ellipses) clustered on the negative PC1 side while limited irrigation samples (I_425_; green, grey, light-grey ellipses) distributed along positive PC1, demonstrating irrigation regime as the primary source of trait differentiation, and three potassium levels formed distinct subgroups within identical irrigation regimes, revealing potassium supply induced secondary trait variation along PC2. Trait loading vectors further revealed consistent multivariate grouping patterns: carbohydrate metabolic enzymes (SS, SPS, β-1,3-Gase), nitrogen metabolism indicators (NR, GS), cell wall carbon compounds (β-1,3-glucan, cellulose), fiber biomass, lint percentage (LP), lint yield (LY), seed cotton yield (SY) and IWUE shared strong positive loadings on PC1 and oriented toward the positive PC1 quadrant, free amino acids (FAA) and soluble protein showed positive loadings on both PC1 and PC2, the S/P ratio exhibited positive PC1 but negative PC2 loadings with its vector opposing FAA and soluble protein along the PC2 axis, and cellulose displayed exclusive negative PC2 loading and moderate negative PC1 loading that separated it from most metabolic and yield-related traits. Among all treatments, I_425_K_2_ occupied the extreme positive PC1 region and aligned with loading vectors of LY, SY, IWUE, fiber biomass, LP, β-1,3-glucan and β-1,3-Gase, whereas I_500_K_1_ positioned at the negative PC1 and positive PC2 end and closely matched the cellulose vector, and low-potassium treatments (K_1_) deviated farthest from yield and water-use efficiency vectors under both irrigation supplies.

## 4 Discussion

### 4.1 Water-potassium interactive regulation of fiber biomass, cellulose biosynthesis and lint productivity

Drought represents the dominant abiotic barrier limiting lint yield and quality in arid cotton agroecosystems (Zhang et al., 2022), while potassium acts as a pleiotropic metabolic modulator to counteract water deficit via osmotic homeostasis and enzyme activation (Daoud et al., 2020; Zahoor et al., 2017b). Crop productivity arises from the interplay of genotype, environment and agronomic management (Patil et al., 2023), and our three-year field trial disentangled the context-dependent water-potassium interactive effects on lint formation across interannual climatic fluctuations. We detected divergent irrigation responses under K-limited supply: full irrigation (I_500_) only elevated lint yield in 2021 under K_1_, an ephemeral benefit driven by enhanced root water uptake (Li et al., 2024), yet chronic K depletion impaired systemic drought acclimation across subsequent growing seasons (Loka et al., 2019). For moderate and high potassium regimes, limited drip irrigation (I_425_) boosted lint output markedly in 2022, yet irrigation-mediated yield gaps narrowed substantially in 2023. This interannual divergence can be directly attributed to the meteorological conditions shown in **Figure 1**: the 2022 growing season had moderate temperatures and low late-season rainfall (total seasonal precipitation 153 mm; cumulative temperature ≥15 °C of 3175 °C·d), which preserved the soil moisture gradient between I_425_ and I_500_ and allowed K-mediated physiological compensation to fully express. In contrast, 2023 was characterized by delayed sowing (May 2 vs. April 24), lower cumulative temperature (2962 °C·d) and substantially higher late-season rainfall (189 mm); spring chilling suppressed root K uptake capacity, while abundant post-boll precipitation replenished soil water in the I_500_ plots and effectively erased the mild water-deficit signal that drives the K compensation effect (Fu et al., 2023). Thus, the climatic context directly modulates the magnitude of water–potassium synergy on lint yield. Interannual plasticity of water-potassium synergy underscores that biomass partitioning to reproductive sinks is highly climate-dependent (Ding et al., 2023), and cool springs plus post-boll rainfall blunt root nutrient-water capture gradients, eroding yield disparities on lower canopy fruiting branches. Consistent with prior cotton nutrition work (Ali et al., 2018; Hafeez et al., 2019), potassium supply positively modulated lint yield, yet dose responses diverged sharply by irrigation regime. Under full irrigation, lint yield scaled linearly with K input, as abundant water facilitated translocation of photoassimilates to fiber sinks. Under limited drip irrigation, yield followed a unimodal parabolic curve peaking at K_2_. This attenuation at high K may involve K^+^-induced cation imbalance (e.g., reduced Ca^2+^ or Mg^2+^ availability) that could impair cell wall maturation (Wakeel, 2013), although tissue mineral concentrations were not measured and this interpretation remains speculative. Even in the cooler 2023 growing season, K_2_ maintained a 15.0% total lint gain relative to K_1_, while K_3_ efficacy declined, attributable to seedling cold stress depressing root K acquisition and late-stage K leaching; this confirms moderate potassium confers superior physiological robustness under combined water and temperature stress.

Assimilate partitioning between seed and fiber dictates harvest index, with lint percentage acting as a core partitioning metric (Chen et al., 2018). Drought restricts photoassimilate translocation to bolls and suppresses fiber biomass accrual (Zhao et al., 2019), but potassium rewires sink-source allocation to prioritize economic fiber tissue. Under limited drip irrigation, K_2_ consistently elevated fiber biomass fraction relative to K_1_, supporting the K-mediated preferential sink allocation model (Hu et al., 2016), wherein K stimulates fiber cell elongation to shift dry matter toward harvestable tissue. Because extended rapid biomass accumulation incurs higher respiratory loss (Andrade et al., 2022), K_2_ strikes a balance between accumulation duration and carbon retention, which translates into a higher lint percentage across limited drip irrigation treatments. This fiber partitioning shift may be related to K^+^-mediated enhancement of cellulose synthesis, possibly through activation of cellulose synthase (CesA) complexes; however, CesA activity or expression was not measured in the present study, and this hypothesis requires direct molecular verification (Chen et al., 2023).

Water stress remodels fiber biomass growth trajectories: upper canopy bolls initiate rapid accumulation prematurely as an adaptive reproductive prioritization strategy (Leffler, 1976), yet this temporal advance truncates the rapid-growth window and depresses theoretical maximum boll biomass, representing a canonical stress fitness trade-off (He et al., 2015). Canopy vertical stratification generates divergent stress susceptibility: lower branches face simultaneous water and light limitation, cutting theoretical maximum fiber biomass by 11.0% under deficit irrigation (Chen et al., 2018). Potassium eliminates this canopy disparity: under limited drip irrigation, K_2_ markedly boosting the maximum fiber accumulation rate and achieves a theoretical peak cotton boll biomass that outperforms conventional drip irrigation counterparts, demonstrating that moderate K can fully compensate for mild water deficit in fiber sink strength. This stress compensation aligns with the optimal K concentration framework moderate K may sustain cellular osmotic buffering without inducing the K^+^-divalent cation antagonism that could impair wall development at high K inputs.

Potassium may also modulate fiber developmental physiology through hormonal pathways; for instance, altered gibberellin and ethylene signaling has been associated with K-mediated fiber development in previous studies (Hu et al., 2015), although hormone concentrations were not quantified here. All gains in fiber biomass and lint fraction trace back to enhanced cellulose deposition, the structural polymer comprising >90% of mature fiber dry weight and our Mantel correlation and PCA results (**Figures 8**, **9**) revealed strong positive multivariate associations between cellulose abundance, fiber biomass and lint yield (Mantel’s r ≥ 0.5, P < 0.001). Drought suppresses sucrose-metabolizing enzymes, curtails UDPG precursor pools and shunts carbon toward callose synthesis, lowering cellulose deposition we recapitulated this phenotype in K_1_ treatments, while K_2_ and K_3_ rescued cellulose production, with the largest gains realized under I_425_. The protective effect of K on lint yield is fully mediated by elevated cellulose content, yet climatic variability modulates water-K efficacy: 2023 spring cold and late precipitation blunted treatment differences, illustrating that multi-year field phenotyping is indispensable to design climate-resilient cotton fertigation regimes (Fu et al., 2023).

**Figure 9:**
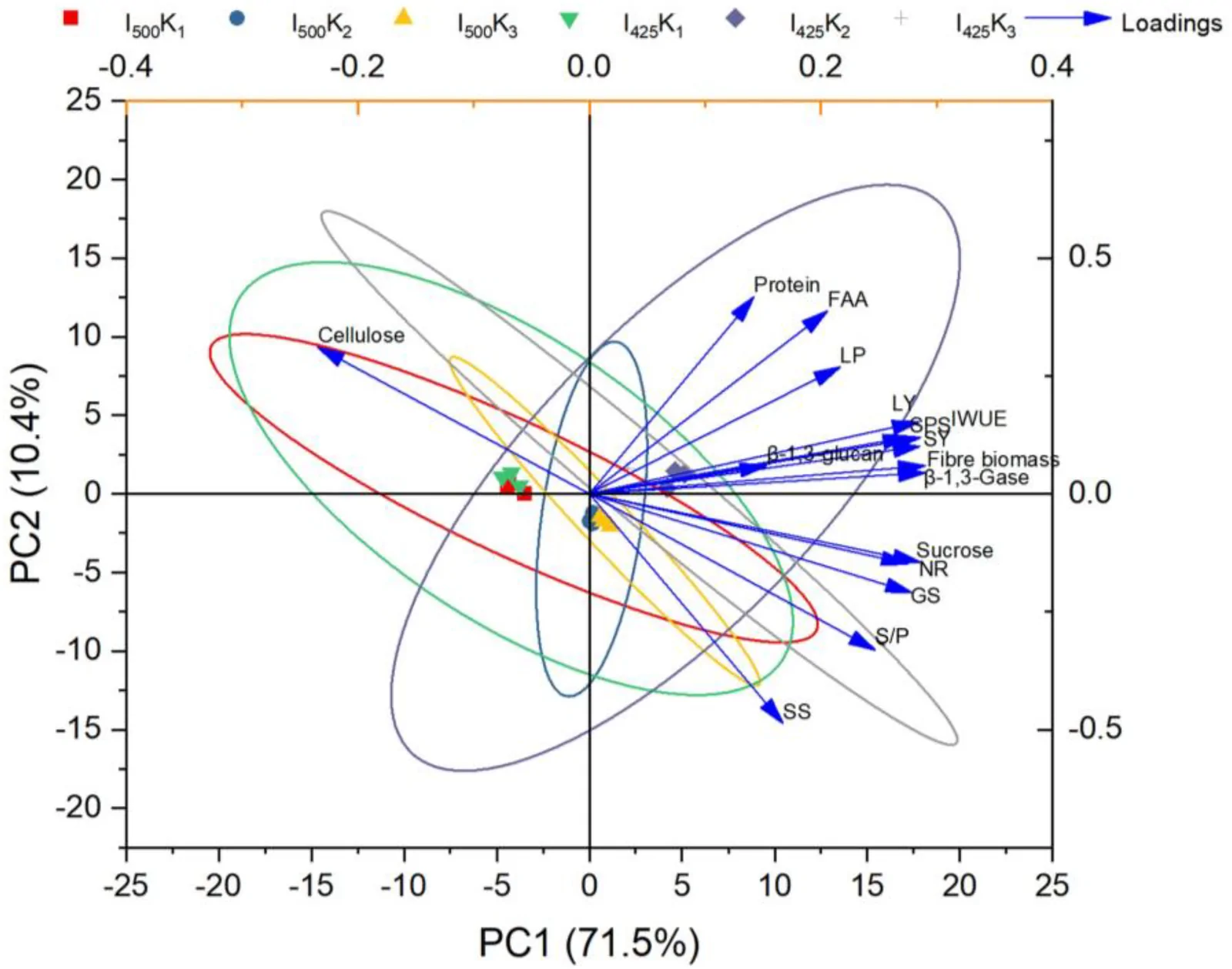
Principal component analysis (PCA) of cotton physiological, yield and water-use efficiency traits across all irrigation–potassium treatments. Ellipses represent 95% confidence intervals for each treatment group. Different lowercase letters indicate significant differences at P < 0.05.

Collectively, the I_425_K_2_ fertigation regime achieves a sustainable production trade-off: it accelerates fiber biomass accumulation, extends active developmental windows, counteracts drought-induced cellulose suppression, and simultaneously elevates lint yield and irrigation water use efficiency (IWUE), rendering it a climate-smart agronomic solution for water-scarce arid cotton zones.

### 4.2 Medium potassium coordinates fiber carbon and nitrogen-metabolic pathways to boost cellulose synthesis under limited drip irrigation

Sucrose and β-1,3-glucan constitute the core carbon precursors fueling cellulose polymerization (Chantal et al., 2007; Zhao et al., 2020). Drought blocks leaf-to-boll sucrose translocation and depletes fiber soluble carbon pools during early elongation (20–25 DPA) (Zhao et al., 2019), a defect fully rescued by potassium supplementation. Under deficit irrigation, K_2_ and K_3_ substantially elevated fiber sucrose and β-1,3-glucan concentrations at 20–30 DPA, driven by enhanced activity of three carbon metabolic rate-limiting enzymes: SS, SPS and β-1,3-glucanase (Hu et al., 2017). Notably, in the I_425_K_2_ treatment, fiber sucrose content declined during 40–50 DPA while cellulose content increased sharply (**Figures 5**, **10**). This inverse temporal pattern reflects a substrate-to-product conversion rather than a carbon shortage: during secondary wall thickening, highly activated SS (**Figure 11**) cleaves sucrose into UDPG and fructose, and β-1,3-glucanase simultaneously degrades callose to release additional glucose equivalents (Tucker et al., 2001; Hu et al., 2022). The resulting UDPG flux is directly channeled to cellulose synthase complexes for rapid microfibril polymerization. Thus, the simultaneous elevation of SS and β-1,3-glucanase activities under I_425_K_2_ accelerates sucrose turnover, causing the soluble sucrose pool to decrease while cellulose deposition is maximized at 40–50 DPA. SS and SPS catabolize sucrose to generate UDPG monomers, while β-1,3-glucanase degrades callose to release additional carbon skeletons; together they sustain the high UDPG flux demanded for rapid cellulose deposition (Hu et al., 2022). Water deficit suppresses all three enzymes, interrupting carbon cycling and limiting polymer formation (Gao et al., 2019; Tang and Xiao, 2013). As a universal enzyme cofactor (Loka et al., 2019), K counteracts drought-mediated enzyme inhibition; among all treatments, I_425_K_2_ triggers the most pronounced activation of SS, SPS and β-1,3-glucanase across boll developmental stages (detailed in **Figure 11**), confirming that medium K is the optimal dose to restore carbon flux under mild water stress. The combined carbon substrate surplus directly elevates cellulose pools (Kumar et al., 2020). Critically, K-mediated carbon metabolic stimulation is far more pronounced under limited irrigation than full water supply, and K_2_ outperforms K_3_ in sustaining sucrose turnover, confirming medium potassium as the optimal dose to restore carbon flux under mild water stress.

**Figure 10:**
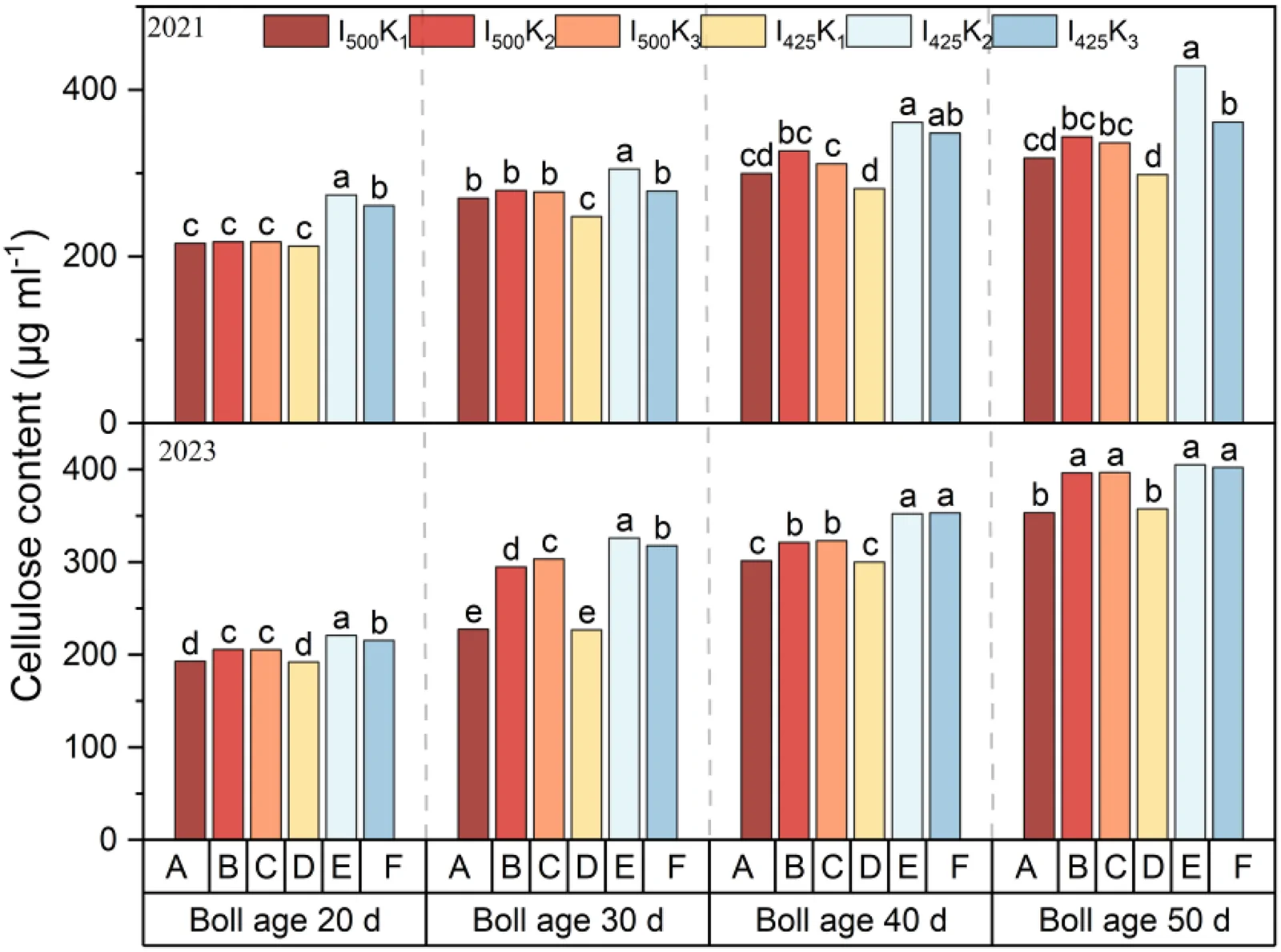
Effects of irrigation regime and potassium fertilization on cotton cellulose content (2021–2023). Different lowercase letters within the same boll age indicate significant differences among treatments at P < 0.05 according to Duncan’s multiple range test (n = 3).

**Figure 11:**
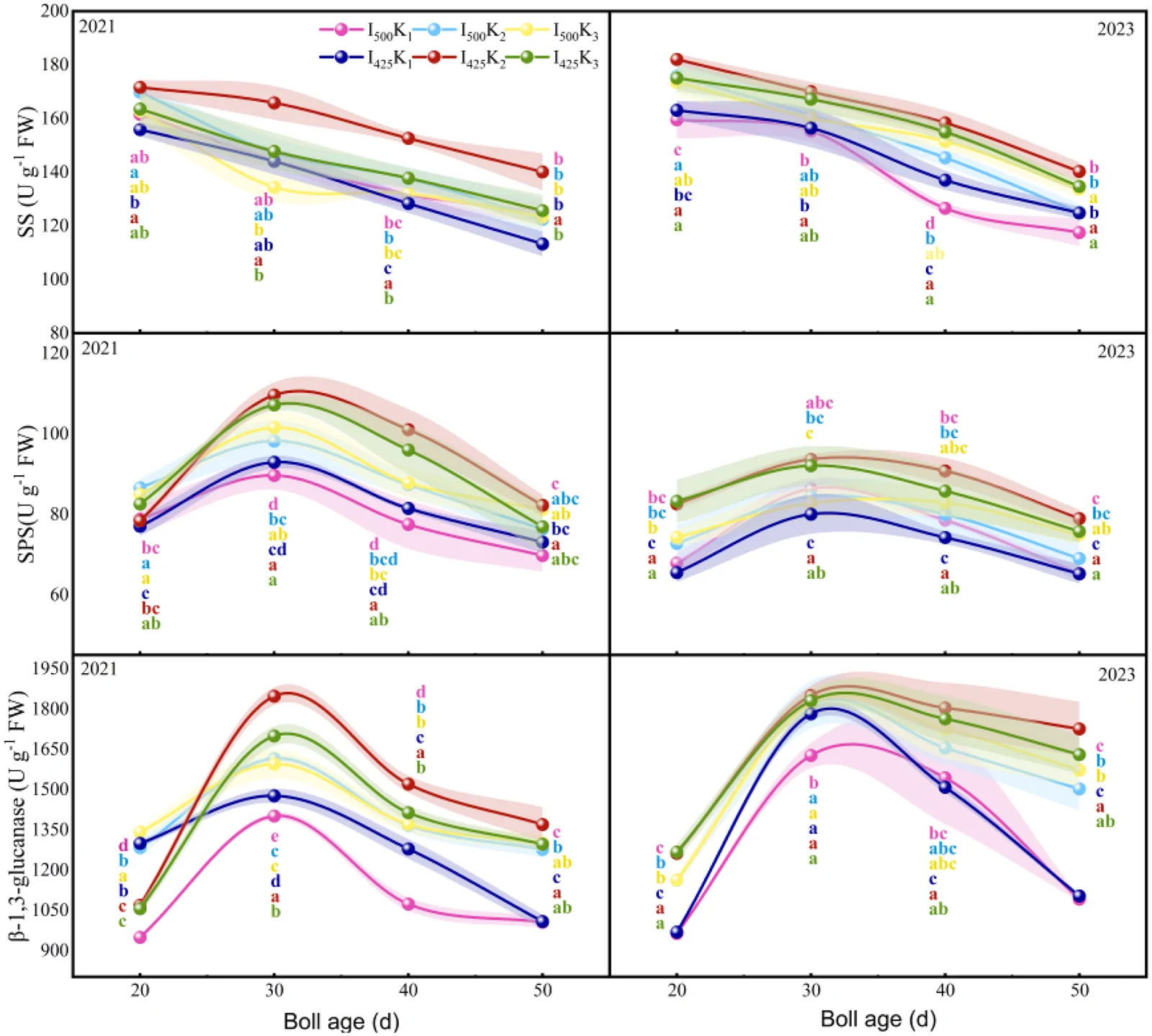
Effects of irrigation regime and potassium fertilization on the activities of key enzymes in cotton fiber carbon metabolism (2021 and 2023). Different lowercase letters within the same boll age indicate significant differences among treatments at P < 0.05 according to Duncan’s multiple range test (n = 3). SS, sucrose synthase; SPS, sucrose phosphate synthase activity.

Our physiological data suggest that I_425_K_2_ coordinates carbon and nitrogen enzyme activities to support fiber elongation and secondary wall thickening. Distinct K effects on nitrogen pools emerged across irrigation regimes: K_2_ elevated fiber FAA under limited drip irrigation but suppressed FAA under conventional drip irrigation. This irrigation-dependent reversal of the FAA response to K represents a key finding of this study. Under conventional drip irrigation, water is not limiting and K-mediated activation of NR and GS (**Figure 4**) accelerates the conversion of FAA into soluble protein for cell expansion and structural protein synthesis (**Figure 6**), thereby depleting the FAA pool. In contrast, under limited drip irrigation, mild water deficit induces osmotic adjustment in fiber cells; K^+^ promotes the accumulation of free amino acids (e.g., proline and glutamine) as compatible osmolytes to maintain cell turgor, while concurrently activated NR/GS continuously supplies newly synthesized amino acids. Because the rate of amino acid accumulation for osmotic protection exceeds its consumption for protein synthesis under water deficit, the FAA pool is elevated. This dual function of K—osmotic stabilization under drought and nitrogen assimilation activation—underlies the contrasting FAA patterns between the two irrigation regimes. In addition, this pattern arises from the dual functions of K under drought: osmotic stabilization and NR/GS activation jointly drive root nitrogen uptake and nitrate-to-amino acid conversion (Chantal et al., 2007; Gandhamanagenahalli et al., 2024), and the resulting FAA pool fuels soluble protein synthesis and cell wall loosening proteins production (Wang et al., 2022)fiber (Wang et al., 2022). Optimized coordination between carbon substrate supply and nitrogen assimilation under K_2_ enhances cellulose by 22.7% relative to I_500_K_1_ via three interconnected pathways: (1) GS-derived glutamine may support the biosynthesis of cellulose synthase complexes (Tucker et al., 2001); (2) K^+^ may activate SS (possibly through allosteric regulation), which could enhance the supply of UDP-glucose precursors for cellulose synthesis (Wang et al., 2019); consistent with this, we observed elevated SS activity and sucrose content in I_425_K_2_ fibers, although UDP-glucose was not directly quantified; (3) GS-mediated nitrogen assimilation reroutes fixed carbon away from defensive secondary metabolism toward cellulose deposition (Zahoor et al., 2017b). Excess K_3_ may disrupt this balance via K^+^-Ca^2+^/Mg^2+^ antagonism (Han et al., 2023); given that Mg^2+^ serves as a cofactor for cellulose synthesis (Fujii et al., 2010), K^+^-induced Mg^2+^ limitation could partly explain the attenuated cellulose gains under high potassium, although this remains to be verified by tissue mineral analysis. Developmental stage partitioning of C-N demand underpins this synergy: elongation (20–30 DPA) prioritizes nitrogen for cell expansion, while secondary wall thickening (30–50 DPA) depends on carbon-dominated cellulose polymerization (Tang et al., 2022).

The fiber S/P ratio may serve as a useful auxiliary indicator linking K nutrition, cellulose synthesis and final lint productivity (Ali et al., 2018). Irrigation volume alone exerted no significant effect on the S/P ratio, yet the water–potassium interaction strongly modulated this ratio during secondary wall thickening (40–50 DPA). K_1_ fibers exhibited an elevated S/P ratio, likely reflecting impaired nitrogen assimilation rather than enhanced carbon flux: carbon import continued under K limitation, but insufficient nitrogen reserves may have restricted CesA and structural protein synthesis, leaving available carbon substrates underutilized for cellulose polymerization. By contrast, I_425_K_2_ maintained a low fiber S/P ratio, consistent with more synchronized carbon catabolism and nitrogen assimilation that may channel photoassimilates toward cellulose production. Our PCA and Mantel tests (**Figures 8**, **9**) corroborated this: C/N ratio exhibited strong negative correlations with cellulose, fiber biomass and lint yield, indicating that a low S/P ratio was associated with higher cellulose deposition and lint yield.

This cascade of K-triggered coordination between carbon and nitrogen metabolism translates to agronomic output: I_425_K_2_ delivered the three-year maximum total lint yield, driven by synchronized activation of carbon (SS, SPS, β-1,3-glucanase) and nitrogen (NR, GS) enzymatic machineries. This dual activation secures ample nitrogen cofactors for CesA biosynthesis and continuous UDPG carbon supply for cellulose polymerization throughout secondary wall formation. High K_3_ fails to match K_2_ performance due to cation antagonism limiting Ca^2+^ and Mg^2+^ uptake, highlighting the necessity of balanced, moderate potassium fertigation to synchronize fiber carbon and nitrogen metabolism under arid limited drip irrigation.

Although the proposed mechanisms involving CesA activation, hormonal regulation, and cation antagonism are consistent with our physiological data, direct molecular and elemental evidence would further strengthen these interpretations. Future studies could focus on quantifying UDP-glucose concentrations, cellulose synthase (CesA) activity and gene expression, tissue K, Ca, and Mg concentrations, as well as hormonal levels. In addition, transcriptomic, proteomic, and metabolomic analyses would be valuable for unraveling the regulatory networks underlying these responses. Such investigations will help to validate and refine the mechanistic models presented here.

## 5 Conclusion

Our findings establish a functional water-potassium regulatory framework for arid cotton cultivation (**Figure 12**). Regulated deficit irrigation (425 mm) combined with medium potassium application (144 kg K_2_O ha^-1^) co-activates fiber carbon- and nitrogen-metabolic rate-limiting enzymes to optimize the coordination between carbon substrate supply and nitrogenous metabolite availability during secondary wall thickening. Such metabolic coordination increases carbon precursor contents, enhances cellulose deposition by 22.7% relative to I_500_K_1_, and persistently elevates fiber biomass, lint yield and irrigation water use efficiency over three years. Mantel correlation and PCA (**Figures 8**, **9**) showed that coordinated carbon/nitrogen enzyme activity, a low S/P ratio, and high cellulose content were associated with cotton productivity. I_425_K_2_ outperforms both low-K and high-K treatments; excess potassium may induce cation imbalance that potentially limits cellulose synthesis, while potassium shortage disrupts the sucrose-to-protein balance and assimilate allocation. This study reveals a previously unreported synergistic pathway whereby potassium coordinates fiber carbon- and nitrogen-metabolic pathways under moderate drought, delivers practical fertigation strategies for water-limited oasis cotton regions, and offers a physiological basis for climate-adaptive cotton production under global water scarcity.

**Figure 12:**
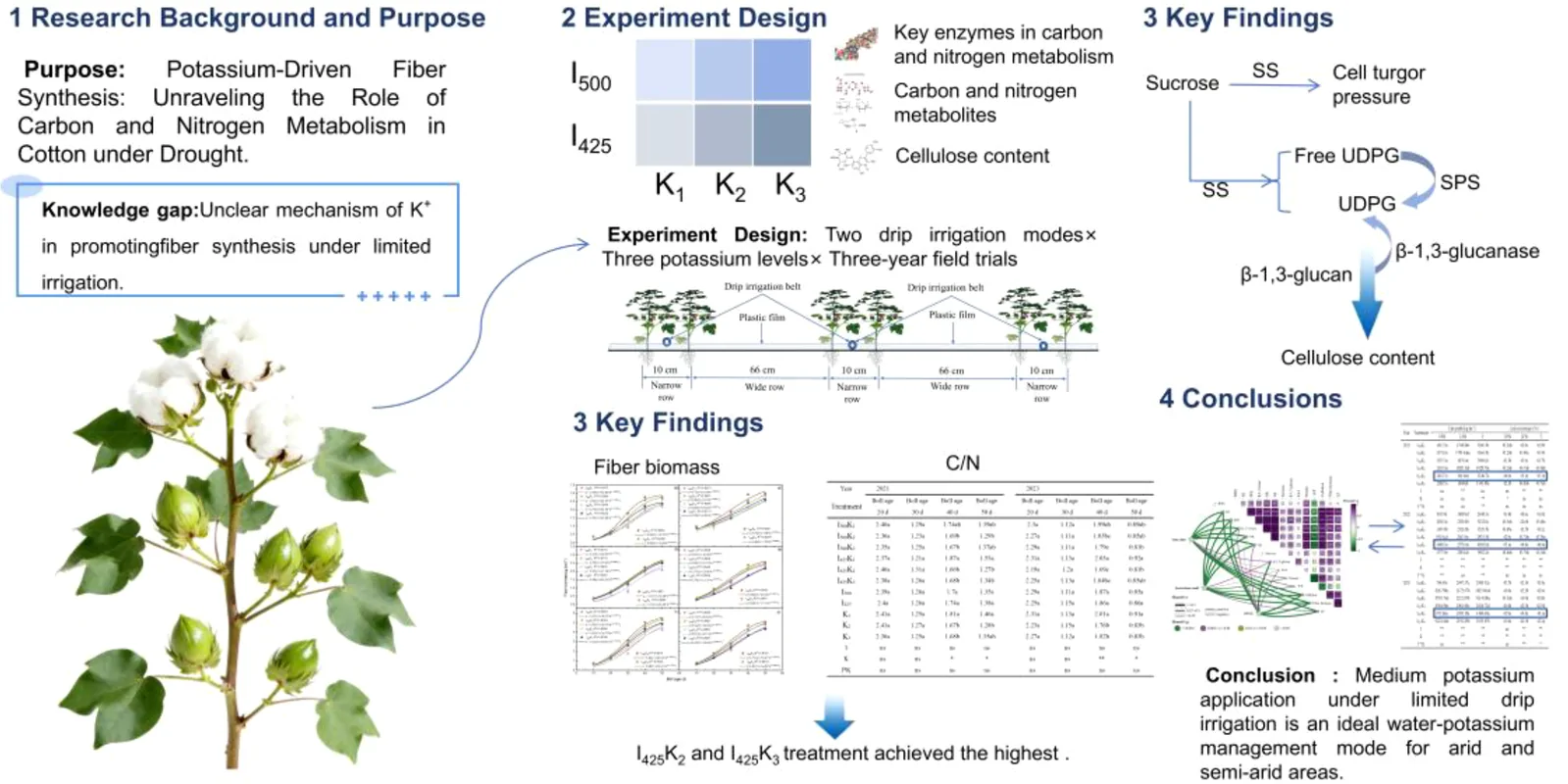
Visual summary of the research.

## Data Availability

The original contributions presented in the study are included in the article/supplementary material. Further inquiries can be directed to the corresponding author/s.
